# Mitochondrial Fission Regulator 1-Like Protein Protects the Heart from Ischemia/Reperfusion Injury via Dual Mitochondrial Mechanisms

**DOI:** 10.34133/research.1241

**Published:** 2026-04-13

**Authors:** Mingyu Wei, Yuanxiu Song, Min Zhu, Xianjing Hu, Shuhong Ma, Shuai Zhang, Pengxin Xie, Rui Guo, Zhen Ma, Xiaojie Hou, Siyao Zhang, Jiaqi Fan, Xian Yang, Amina Saleem, Feng Lan, Xujie Liu, Ming Cui

**Affiliations:** ^1^ Department of Cardiology and Institute of Vascular Medicine, Peking University Third Hospital, Beijing 100191, China.; ^2^ State Key Laboratory of Vascular Homeostasis and Remodeling, NHC Key Laboratory of Cardiovascular Molecular Biology and Regulatory Peptides, Peking University, Beijing 100191, China.; ^3^ Department of Emergency Medicine, The First Affiliated Hospital, Zhejiang University School of Medicine, Zhejiang 310003, China.; ^4^ State Key Laboratory of Cardiovascular Disease, Key Laboratory of Pluripotent Stem Cells in Cardiac Repair and Regeneration, Fuwai Hospital, National Center for Cardiovascular Diseases, Chinese Academy of Medical Sciences and Peking Union Medical College, Beijing 100037, China.; ^5^ Nursing Department, Peking University Third Hospital, Beijing, China.; ^6^ Center for Cardiac Intensive Care, Beijing Anzhen Hospital, Capital Medical University, Beijing 100029, China.; ^7^ Department of Cardiac Surgery, Peking University Third Hospital, Beijing 100191, China.; ^8^ Heart Failure and Valve Surgery Center, Beijing Anzhen Hospital, Capital Medical University, Beijing 100029, China.; ^9^ Department of Cardiovascular Surgery and Institute of Cardiovascular Surgery, West China Hospital, Sichuan University, Chengdu 610041, Sichuan, China.; ^10^ Maternal-Fetal Medicine Center in Fetal Heart Disease, Capital Medical University, Beijing Anzhen Hospital, Beijing 100029, China.; ^11^ Beijing Institute of Heart, Lung, and Blood Vessel Diseases, Anzhen Hospital, Capital Medical University, Beijing 100029, China.; ^12^ State Key Laboratory of Cardiovascular Disease, Fuwai Hospital Chinese Academy of Medical Sciences, Shenzhen, Guangdong, China.

## Abstract

Mitochondrial dysfunction is pivotal in the pathogenesis of cardiac ischemia/reperfusion (I/R) injury. Restoring mitochondrial function represents a promising strategy for mitigating I/R-induced cardiac injury. Mitochondrial fission regulator 1-like protein (MTFR1L), a recently identified mitochondrial dynamics protein, is abundantly expressed in the cardiac tissues. However, its functional role in I/R injury remains undefined. Here, Mtfr1l-knockout mice and human embryonic-stem-cell-derived cardiomyocytes are utilized to investigate the role of MTFR1L in myocardial I/R injury and elucidate its contribution to mitochondrial integrity and function. MTFR1L deficiency markedly worsened I/R-induced cardiac injury and mitochondrial dysfunction. These phenotypes were partially reversed by mitochondria-anchored apoptosis-inducing factor (AIF) overexpression. Mechanistically, MTFR1L protects the heart via 2 interconnected pathways. First, MTFR1L sustains AIF dimerization and stabilizes the AIF–CHCHD4 (coiled-coil-helix-coiled-coil-helix domain containing 4) complex, thereby preserving mitochondrial contact site and cristae organizing system integrity and cristae architecture to facilitate electron transport chain supercomplex assembly, sustain mitochondrial respiration, and limit reactive oxygen species production. Second, by physically interacting with AIF, MTFR1L prevents its mitochondrial release and nuclear translocation, thereby suppressing intrinsic apoptosis. Overall, these findings identify MTFR1L as a cardioprotective protein against myocardial I/R injury through a dual mechanism, providing new insights into the functional repertoire of MTFR1L beyond its previously recognized role in mitochondrial dynamics. Targeting MTFR1L or its interactors may offer novel therapeutic strategies for alleviating mitochondrial dysfunction and myocardial injury.

## Introduction

Acute myocardial infarction (AMI), a leading cause of global mortality and heavy economic burden [1], is primarily treated with percutaneous coronary intervention (PCI) to restore coronary blood flow. However, while timely reperfusion is crucial for limiting infarct size, it paradoxically induces additional myocardial damage through ischemia/reperfusion (I/R) injury, a critical unresolved challenge in AMI management due to incomplete understanding of its mechanisms and the persistent lack of effective preventive strategies [2].

Previous studies have shown that bioenergetics disturbances, oxidative stress, calcium overload, and apoptosis are all major contributors to cardiomyocytes death in myocardial I/R injury [3]. Central to these processes, mitochondria, as the powerhouse of the heart, are recognized as key triggers of cell death in I/R injury [4,5]. To adapt to myocardial hypoxia (ischemia), the mitochondrial function dramatically alters, primarily through the profound suppression of oxidative phosphorylation (OXPHOS) and a drastic reduction in adenosine triphosphate (ATP) production. Upon reperfusion, the sudden influx of oxygen overwhelms the compromised electron transport chain (ETC), leading to an outbreak of oxidative stress within the mitochondria. This oxidative burst damages mitochondrial DNA and associated membrane structures. Beyond this, a cascade of interconnected mitochondrial dysfunction during I/R, including ATP depletion, mitochondrial Ca^2+^ overload, mitochondrial permeability transition pore opening, and the release of cytochrome c, acts synergistically to drive cell death [6,7]. Therefore, strategies aimed at preserving proper mitochondrial structural integrity and functional capacity, collectively referred to as mitochondrial homeostasis, to counteract I/R-induced damage represent a promising therapeutic avenue for treating cardiac I/R injury [8,9].

Mitochondrial quality control serves as the fundamental mechanism to maintain mitochondrial homeostasis under both physiological and pathological conditions. The mitochondrial quality control network consists multiple interacted process, including mitochondrial dynamics (specifically referred as fusion and fission), mitophagy, biogenesis, proteostasis, and the mitochondrial unfolded protein response, all of which cooperate to ensure mitochondrial integrity and function [10,11].

Mitochondrial fission regulator 1-like (MTFR1L), a recent discovered substrate of adenosine-monophosphate-activated protein kinase (AMPK) [12], has been shown to be a novel mitochondrial dynamics regulator. It belongs to the MTFR1 family, which includes MTFR1 and MTFR2 [13]. Both of them have been shown to regulate mitochondrial structure and bioenergetics [14,15]. Tissue specific genome-wide transcriptomics and proteomics analyses have revealed a high expression level of MTFR1L in the heart [16], underscoring its potential relevance to cardiac physiology. Moreover, MTFR1L has been demonstrated to localize to the outer mitochondrial membrane, where it regulates mitochondrial morphology by promoting fragmentation through antagonism of the profusion protein optic atrophy 1 [OPA1] [12,17]. Based on these findings, we hypothesize that MTFR1L may play a crucial role in maintaining mitochondrial homeostasis during myocardial I/R injury.

In this study, we investigated the potential contribution of MTFR1L in cardiac I/R injury using both human embryonic-stem-cell-derived cardiomyocytes (hESC-CMs) and an Mtfr1l gene knockout (Mtfr1l-KO) transgenic mouse model. Our findings reveal a cardioprotective role of MTFR1L during I/R injury, as its genetic suppression notably exacerbated cardiac I/R injury, evidenced by increased mortality, worsened cardiac function, and larger infarct and fibrotic areas compared to I/R treatment alone. These detrimental effects are likely attributable to severe disruption of mitochondrial homeostasis, including cristae disorganization, impaired energy production, and elevated oxidative stress.

Mechanistically, our study reveals that MTFR1L protects the heart from I/R injury through 2 distinct yet potentially interrelated mechanisms. First, MTFR1L preserves mitochondrial cristae structure to support the assembly and enzymatic activity of individual ETC complexes and supercomplexes, thereby maintaining mitochondrial bioenergetics and limiting excessive reactive oxygen species (ROS) generation. Second, MTFR1L tethers apoptosis-inducing factor (AIF) within mitochondria, restricting its mitochondrial-to-nuclear translocation and thus suppressing intrinsic apoptosis in cardiomyocytes during myocardial I/R stress. Notably, In vivo overexpression of mitochondria-anchored AIF (Mt-AIF) either before or post-I/R can mitigate cardiac injury. These data not only unveil the dual role of MTFR1L in safeguarding the cardiomyocytes from I/R injury but also underscore its potential as a novel therapeutic target for mitigating myocardial I/R damage.

## Results

### MTFR1L expression is reduced in cardiac I/R

**Fig. 1.**
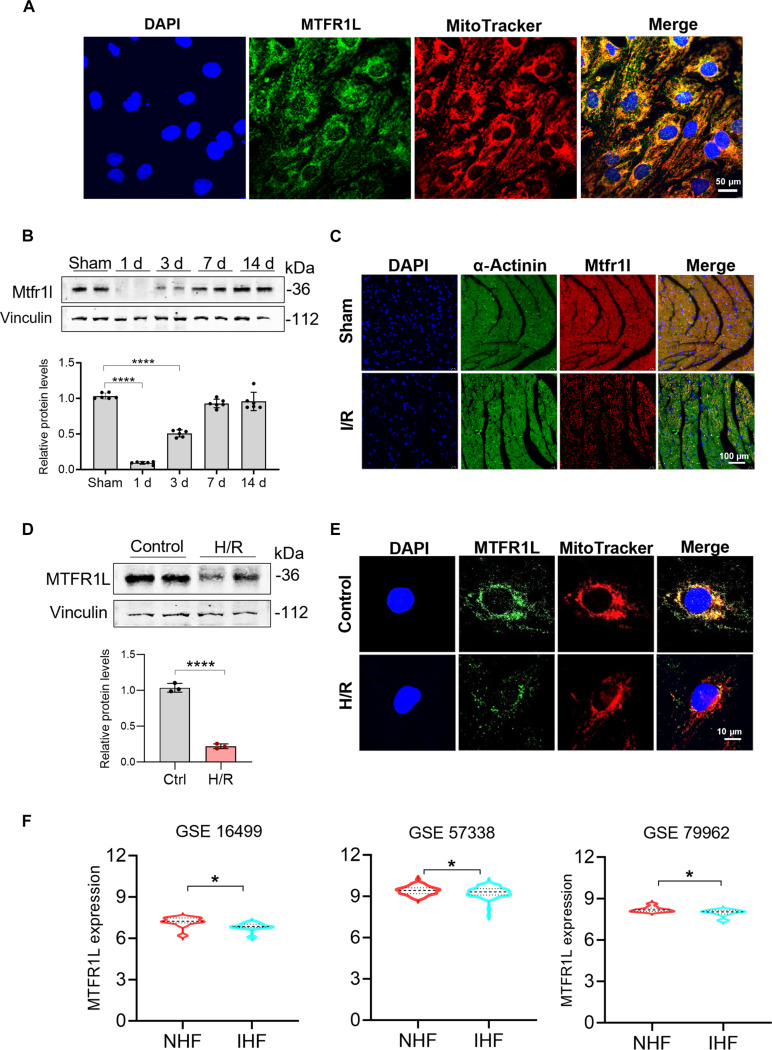
Mitochondrial fission regulator 1-like protein (MTFR1L) expression location and alteration during ischemia/reperfusion (I/R) treatment. (A) Immunofluorescence staining of colocalization of MTFR1L and mitochondria in H9 cell-derived cardiomyocytes (H9-CMs). Scale bar, 50 μm. (B) Representative Western blot and statistical analysis of cardiac Mtfr1l levels in mouse heart at different time points after 30 min of ischemia, followed by reperfusion. *n* = 6 samples per group. (C) Representative immunofluorescence images of cardiac tissue stained for sarcomeric α-actinin (green) and Mtfr1l (red) after I/R. Scale bar, 100 μm. (D) Western blot detection of MTFR1L in H9-CMs subjected to hypoxia/reoxygenation (H/R) (*n* = 3 per group). (E) Immunofluorescence visualization of MTFR1L (green) and mitochondria (MitoTracker, red) in H9-CMs following H/R. Scale bar, 10 μm. (F) Bioinformatic analysis of MTFR1L expression in human ischemic heart failure samples from Gene Expression Omnibus datasets. NHF, nonischemic heart failure; IHF, ischemic heart failure. Data are represented as means ± SEM; *P < 0.05 and ****P < 0.0001. Data were analyzed via unpaired 2-tailed Student’s *t* test (D and F).

We first examined Mtfr1l expression in various mouse tissues. Consistent with previous report [12], Mtfr1l showed highly expressed in cardiac tissue (Fig. S1A). To further investigate the its expression dynamics in cardiac cells, we differentiated H9 cells into cardiomyocytes (H9-CMs) (Fig. S1B to D) and analyzed its expression during the differentiation process. The results showed that MTFR1L expression progressively increased as differentiation advanced (Fig. S1E). This increased expression, combined with its predominant mitochondrial localization (Fig. 1A), suggests that MTFR1L functions as a mitochondrial effector protein critically involved in heart development.

Next, we analyzed Mtfr1l expression in mouse I/R models and found that its expression sharply declined during the early reperfusion phase (1 d postreperfusion) but then gradually returned to near-baseline levels thereafter (Fig. 1B and C). This dynamic expression pattern was similarly observed in serum from patients with AMI following PCI (Fig. S1F). We then examined MTFR1L expression in H9-CMs subjected to hypoxia/reoxygenation (H/R) and analyzed 3 independent Gene Expression Omnibus datasets of human clinical samples. Both experimental and clinical data consistently showed that MTFR1L expression was significantly down-regulated under hypoxic stress (Fig. 1D to F). Taken together, these findings suggested a strong link between MTFR1L expression and cardiac functions, particularly under ischemia conditions.

### MTFR1L deficiency exacerbates myocardial I/R injury

To investigate MTFR1L’s loss of function effect, we generated global Mtfr1l-KO mice using CRISPR–Cas9-mediated deletion of a 2,317-bp genomic region spanning exons 3 to 5 (Fig. S2A). Successful ablation of gene expression was confirmed by Western blot (Fig. S2B). Unexpectedly, Mtfr1l-KO mice exhibited only a mild decrease in cardiac contractile function (Fig. S2C) but otherwise demonstrated relatively normal viability and development, showing no significant differences from wild-type (WT) littermates in long-term survival rate (Fig. S2D), reproduction capacity, body weight, and gross behavior (data not shown) , as well as histological morphology (Fig. S2E), at least up to 18 months of age.

**Fig. 2.**
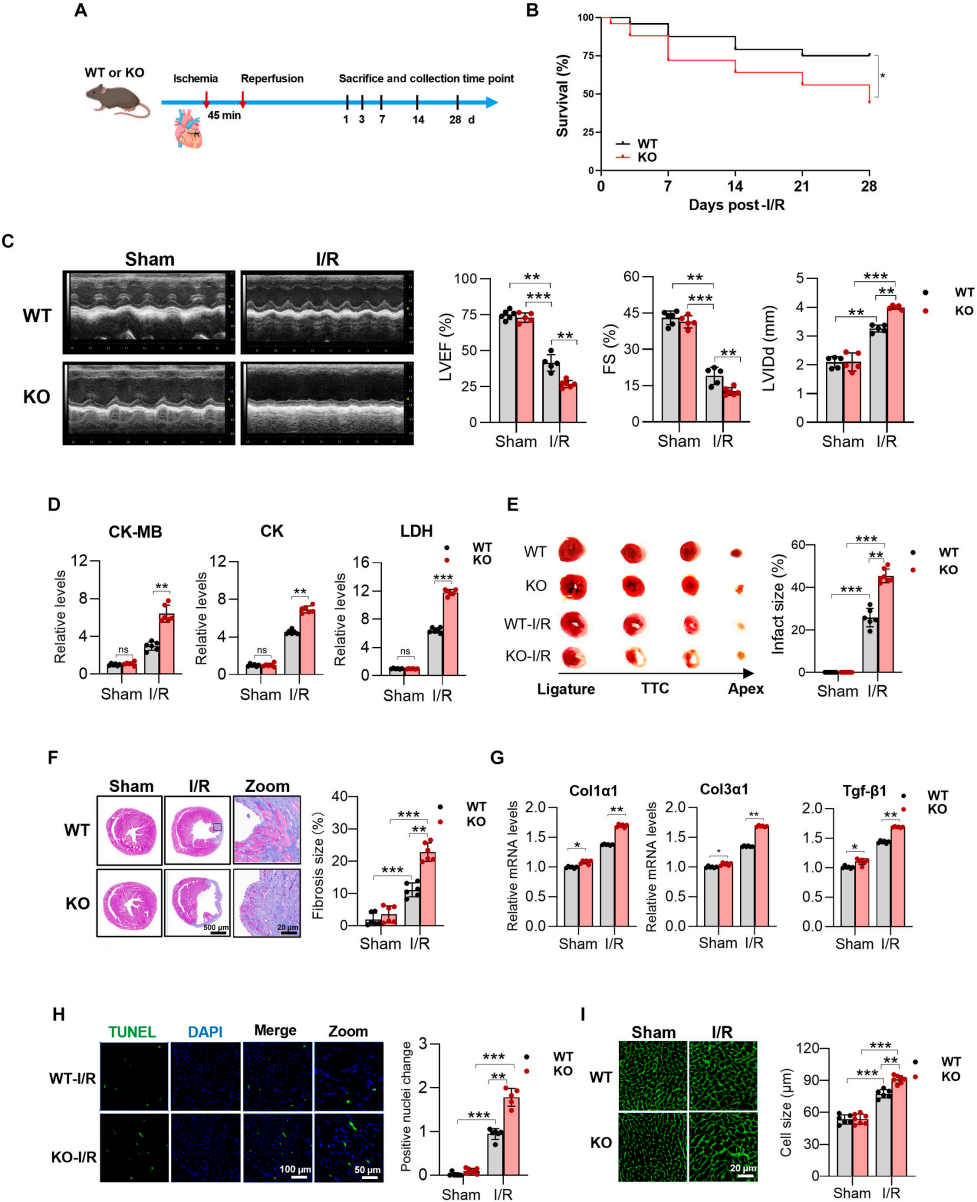
Mitochondrial fission regulator 1-like protein (MTFR1L) deficiency exacerbates cardiac ischemia/reperfusion (I/R) injury. (A) Schematic diagram for evaluating the consequence of MTFR1L deletion following myocardial I/R. (B) Survival curve of Mtfr1l-knockout (KO) and wild-type (WT) mice after I/R. (C) Representative echocardiographic images of Mtfr1l-KO and WT controls at sham (baseline) conditions and under I/R and corresponding quantitative analyses. (D) Serum levels of creatine kinase (CK), CK-myocardial band (CK-MB), and lactate dehydrogenase (LDH) in WT and Mtfr1l-KO mice 8 h after I/R injury. *n* = 6 per group. (E) TTC (2,3,5-triphenyltetrazolium chloride) staining and statistical analysis for cardiac infarction area from Mtfr1l-KO hearts and WT littermates 7 d after I/R. *n* ≥ 6 per group. (F) Masson trichrome staining of myocardial collagen and quantification 28 d after I/R. Scale bars, 500 and 20 μm. (G) Real-time quantitative polymerase chain reaction (RT-qPCR) analysis of fibrosis related genes expression 28 d after I/R. *n* = 6 per group. (H) Terminal deoxynucleotidyl-transferase-mediated deoxyuridine triphosphate nick end labeling (TUNEL) staining and analysis of apoptosis level in mouse heart 7 d after I/R. Scale bars, 100 and 50 μm. (I) Wheat germ agglutinin (WGA) staining of cardiomyocyte cross-sectional area and quantification 28 d after I/R. Scale bar, 20 μm. Data are represented as means ± SEM; *P < 0.05, **P < 0.01, and ***P < 0.001. Data were analyzed via 2-way ANOVA, followed by the Bonferroni post hoc test.

To evaluate the role of MTFR1L in cardiac I/R injury, we established a mouse cardiac I/R model (Fig. 2A). In contrast to sham conditions, Mtfr1l deficiency markedly exacerbated myocardial injury in murine cardiac I/R model. Following I/R surgery, Mtfr1l-KO mice exhibited markedly reduced survival compared to WT littermates, with the survival divergence becoming statistically significant at 4 weeks postprocedure (Fig. 2B). Echocardiographic analysis revealed much more severe cardiac contractile dysfunction in Mtfr1l-KO mice compared to WT mice under I/R treatment (Fig. 2C). Under sham conditions, both WT (gray) and KO (red) mice displayed only minor differences in cardiac performance, with left ventricular ejection fraction (LVEF; 74.91% versus 72.97%), fractional shortening (FS; 43.98% versus 41.38%), and left ventricular internal diameter at end-diastole (LVIDd; 2.09 mm in both groups). Following I/R injury, cardiac function declined markedly in both genotypes, but the impairment was significantly more pronounced in KO mice. Specifically, LVEF decreased by 44.7% in WT (from 74.91% to 41.44%) and by 63.4% in KO mice (from 72.97% to 26.74%). Similarly, FS was reduced from 42.98% to 19.03% in WT (a 55.7% drop) and from 41.38% to 12.60% in KO (a 69.5% drop). In parallel, LVIDd, a marker of ventricular dilation, increased from 2.09 to 3.26 mm in WT and further to 3.99 mm in KO hearts after I/R. These data collectively demonstrate that Mtfr1l-KO mice experience significantly greater functional decline and adverse structural remodeling following I/R injury, underscoring the detrimental impact of the Mtfr1l deficiency on cardiac performance postinjury. In addition, serum levels of cardiac injury biomarkers, including creatine kinase (CK), CK-myocardial band (CK-MB), and lactate dehydrogenase (LDH), were markedly elevated in Mtfr1l-KO mice 3 d postreperfusion compared to WT controls, suggesting the pronounced deterioration of Mtfr1l-KO hearts (Fig. 2D).

Histopathological and immunohistochemical analysis revealed severe cardiac injury in both WT and Mtfr1l-KO hearts following I/R treatment. However, the damage was significantly exacerbated in Mtfr1l-KO, which displayed ​1.76-fold larger infarct size and 2-fold greater fibrotic size compared to WT controls after I/R injury (Fig. 2E and F). These structural deficits correlated with a stronger up-regulation of fibrosis markers (collagen type I α1 [Col1a1], Col3a1, and transforming growth factor-β1 [Tgf-β1]) in Mtfr1l-KO hearts compared to WT controls (Fig. 2G). In addition, Mtfr1l-KO hearts exhibited significantly increased apoptotic cell numbers, up to 1.9-fold higher, as indicated by terminal deoxynucleotidyl-transferase-mediated deoxyuridine triphosphate nick end labeling (TUNEL) staining, and enlarged cardiomyocyte size, up to 1.2-fold greater times, as measured by wheat germ agglutinin (WGA) staining, compared to controls following I/R treatment (Fig. 2H and I). Collectively, these findings demonstrate that MTFR1L deficiency aggravates cardiac I/R injury, leading to enhanced myocardial damage, elevated apoptosis, and detrimental cardiac remodeling.

### MTFR1L deficiency exacerbates H9-CM H/R injury

To validate MTFR1L loss of function in hESC-CMs, we generated an MTFR1L-KO hESC line. This was achieved by designing a single guide RNA (sgRNA) targeting the exon 2 of the MTFR1L gene and introducing a frameshift mutation in H9 cells using CRISPR–Cas9 (Fig. S3A). A homozygous clone with a ​20-bp deletion​ was isolated by fluorescence-activated cell sorting (Fig. S3B), and Western blot result confirmed the complete absence of MTFR1L protein, establishing the ​H9-MTFR1L-KO​ line (Fig. S3C). The top 5 potential off-target regions within gene exons were examined by Sanger sequencing, and no off-target mutations were detected (Fig. S3D).

Cardiomyocytes differentiated from WT H9 or H9-MTFR1L-KO were subjected to H/R. Consistent with the findings from the mouse I/R model, loss of ​MTFR1L exacerbated cardiomyocyte injury compared to WT, with apoptotic cell counts and LDH release ​approximately doubled in MTFR1L-KO cardiomyocytes after H/R treatment (Fig. S4A and B). In addition, H/R-induced up-regulation of cardiac dysfunction markers, including atrial natriuretic factor, natriuretic peptide B, Col1a, and matrix metalloproteinase 9, were all substantially greater in H9-MTFR1L-KO cardiomyocytes than WT, underscoring an increased vulnerability of MTFR1L-KO cardiomyocytes under stress (Fig. S4C and D).

### Loss of MTFR1L exacerbates mitochondrial damage during I/R

**Fig. 3.**
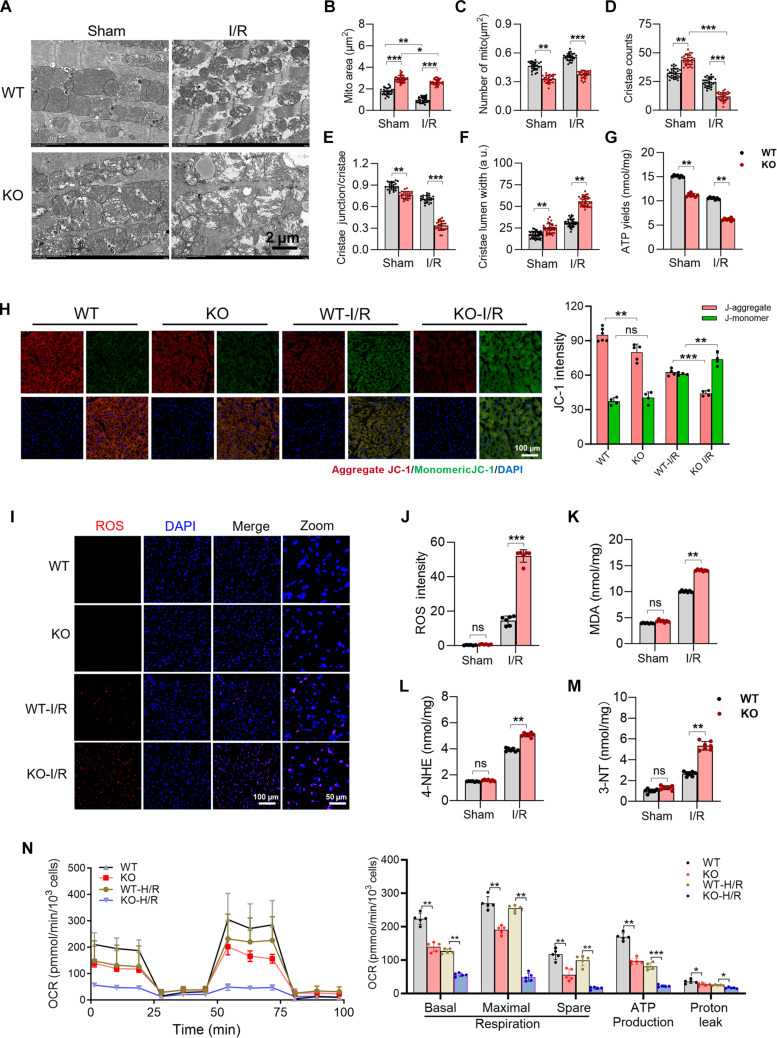
Loss of mitochondrial fission regulator 1-like protein (MTFR1L) exacerbates mitochondrial damage during ischemia/reperfusion (I/R) injury. (A) Representative transmission electron microscopy images showing mitochondrial ultrastructure alterations in heart tissues from Mtfr1l-knockout (KO) and wild-type (WT) mouse under sham or under I/R conditions. Scale bar, 2 μm. (B to F) Quantification of mitochondrial ultrastructural parameters (including size, density, cristae junctions, cristae number, and lumen width). *n* = 6 sample per group. (G) Adenosine triphosphate (ATP) production detection in Mtfr1l-KO and WT cardiomyocytes. *n* = 6 per group. (H) Representative 5,5′,6,6′-tetrachloro-1,1′,3,3′-tetraethylbenzimidazolylcarbocyanine iodide (JC-1) staining images of mitochondrial membrane potential (ΔΨm) in Mtfr1l-KO and WT cardiomyocytes, with corresponding quantification. *n* = 6 per group. Scale bar, 100 μm. (I) Representative fluorescence images of reactive oxygen species (ROS) levels in Mtfr1l-KO and WT cardiomyocytes. Scale bars, 100 and 50 μm. (J) Quantification of ROS levels shown in (I). *n* = 6 per group. (K to M) Quantification of oxidative stress markers (malondialdehyde [MDA], 4-hydroxynonenal [4-HNE], and 3-nitrotyrosine [3-NT]) in heart tissues by immunoassay. *n* =6 per group. (N) Seahorse Mito Stress Test of WT and H9-MTFR1L-KO cell-derived cardiomyocytes (H9-CMs) after hypoxia/reoxygenation (H/R) treatment. Left: Real-time oxygen consumption rate (OCR) profiles. Right: Quantification of key mitochondrial respiration parameters. Data are represented as means±SEM; *P < 0.05, **P < 0.01, and ***P < 0.001. ns, not significant. Data were analyzed via 2-way ANOVA, followed by the Bonferroni post hoc test.

Next, we evaluated the role of MTFR1L in heart mitochondrial quality control under basal conditions and I/R. Transmission electron microscopy results showed that the mitochondrial area in the hearts of Mtfr1l-KO mice increased by 1.7-fold, while the number of mitochondria decreased compared to WT (Fig. 3A to C and Fig. S5A). Similar phenotypes were observed in H9-CMs (Fig. S5B). Such morphological changes are in line with prior studies showing that MTFR1L deficiency leads to mitochondrial fusion [12]. Further analysis of cristae ultrastructure revealed that loss of Mtfr1l significantly increased cristae quantity correlating with an increase in mitochondrial area (Fig. 3A and D and Fig. S5A). However, this was accompanied by a mild reduction in the number of cristae junctions and a slight increase in the width of the cristae lumen (Fig. 3A, E, and F and Fig. S5A). This combination of disrupted organization and compensatory expansion suggests that mitochondrial architecture is both impaired and undergoing actively remodeling to preserve function. To investigate whether these morphological alterations result from changes in canonical mitochondrial dynamics machinery, we examined the expression levels of key regulators including dynamin-related protein 1 (DRP1), mitofusin-1 (MFN1), MFN2, and OPA1. Notably, Western blot analysis revealed a significant increase in OPA1 protein levels in Mtfr1l-KO hearts compared to WT (Fig. S5C), consistent with previous studies linking MTFR1L deficiency to mitochondrial fusion [12]. However, the total protein levels and phosphorylation status of DRP1, MFN1, and MFN2 remained unchanged (Fig. S5C). These findings suggested that the observed mitochondrial fusion phenotype in MTFR1L-deficient hearts is likely attributed to elevated OPA1-mediated membrane fusion.

To elucidate the mechanisms by which Mtfr1l loss exacerbates cardiac dysfunction following I/R injury, we next examined mitochondrial structural and functional alterations under I/R conditions. Compared to WT controls, Mtfr1l-KO cardiomyocytes displayed more pronounced mitochondrial abnormalities after I/R treatment, which underlie the aggravated cardiac dysfunction. During cardiac I/R , WT mice exhibited increased mitochondrial fission, characterized by a decrease in mitochondrial area and an increase in mitochondrial number. In contrast, mitochondria size in Mtfr1l KO mice showed no significant changes (Fig. 3A to C). Mitochondrial swelling even increased from a 1.7-fold elevation at baseline (Mtfr1l-KO versus WT) to 3-fold post-I/R (Fig. 3B). This further underscores that Mtfr1l deficiency impairs mitochondrial fission capacity. Notably, mitochondrial cristae structure in Mtfr1l KO group was further compromised following I/R. Cristae counts of KO group, which were initially elevated to 125% of WT under basal conditions, dropped sharply to 49% after I/R (Fig. 3D). Similarly, cristae junctions were also markedly reduced, declining from 86.7% to 47.4% of WT levels (Fig. 3E), While cristae lumen width of KO group increased from 1.4-fold to 1.8-fold of WT (Fig. 3F). Moreover, numerous low electron-density vacuoles appeared in the mitochondria of KO group (Fig. 3A), which are indicative of mitochondrial swelling or disintegration. These findings demonstrate that Mtfr1l deficiency aggravates I/R-induced mitochondrial injury and underscores its protective role in maintaining cristae architecture under ischemic and oxidative stress.

Mitochondrial function was concurrently impaired in Mtfr1l-KO cardiomyocytes. ATP level of Mtfr1l-KO mitochondria was approximately 74% of WT level under baseline (sham) conditions; however, it further declined to 58% following I/R treatment, indicating that Mtfr1l deficiency exacerbates mitochondrial energy failure under stress (Fig. 3G). Similarly, mitochondrial membrane potential (ΔΨm), assessed by 5,5′,6,6′-tetrachloro-1,1′,3,3′-tetraethylbenzimidazolylcarbocyanine iodide (JC-1) staining, showed a mild reduction in Mtfr1l-KO hearts at baseline. After I/R, both WT and Mtfr1l-KO hearts displayed marked ΔΨm depolarization; however, only in Mtfr1l-KO hearts did the JC-1 monomer signal surpass that of the aggregate form, reflecting a profound loss of mitochondrial polarization (Fig. 3H).

Given that oxidative stress induced by I/R is the primary driver of cardiomyocytes multiple dysfunctions, we next assessed the ROS accumulation and oxidative by-product levels in Mtfr1l-KO hearts. While loss of Mtfr1l did not significantly affect ROS generation under baseline conditions, I/R treatment led to a marked increase in ROS levels in both WT and Mtfr1l-KO cardiomyocytes, with Mtfr1l-KO cells showing up to a 3.5-fold higher accumulation compared to WT (Fig. 3I and J). Oxidative stress was further validated through malondialdehyde (MDA), 4-hydroxynonenal (4-HNE), and 3-nitrotyrosine (3-NT) assays. Consistent with ROS measurement, there were no significant differences between groups under baseline conditions, but I/R significantly elevated the levels of all 3 markers (Fig. 3K to M).

Mitochondrial respiratory capacity was evaluated using Cell Mito Stress Tests (Seahorse assay) in cardiomyocytes derived from H9 and H9-MTFR1L-KO cells. At baseline, MTFR1L deficiency significantly impaired mitochondrial respiration across all measured parameters. This impairment was further exacerbated under H/R stress, with the most pronounced deficits observed in maximal and spare respiratory capacities. Specifically, the WT/MTFR1L-KO ratio for maximal respiration increased from 1.4-fold at baseline to 5.2-fold following H/R, while the ratio for spare respiratory capacity rose from 2.0-fold to 6.2-fold (Fig. 3N).

### MTFR1L supports ETC complex and supercomplex assembly to preserve mitochondrial function during I/R injury

**Fig. 4.**
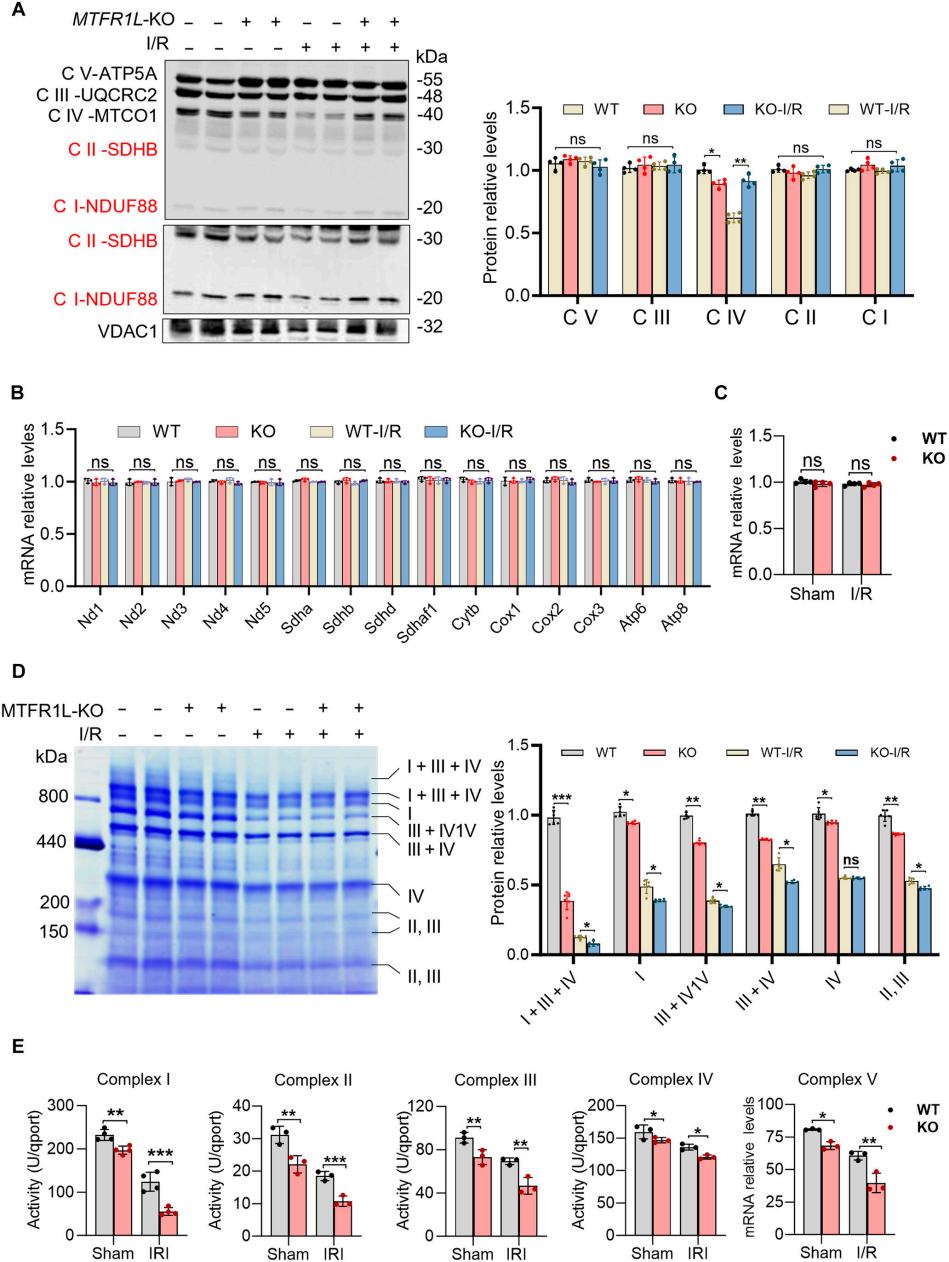
Mitochondrial fission regulator 1-like protein (MTFR1L) regulates electron transport chain (ETC) supercomplex formation. (A) Western blot analysis of representative ETC complex subunits in Mtfr1l-knockout (KO) and wild-type (WT) hearts (left), with corresponding quantification (right). UQCRC2, ubiquinol-cytochrome c reductase core protein 2; MTCO1, mitochondrially encoded cytochrome c oxidase I; SDHB, succinate dehydrogenase complex iron sulfur subunit B; NDUF88, NADH:ubiquinone oxidoreductase subunit S8; VDAC1, voltage-dependent anion channel 1. *n* = 6 per group. (B) Real-time quantitative polymerase chain reaction (RT-qPCR) analysis of additional ETC subunit mRNA levels. *n* = 6 per group. (C) RT-qPCR analysis of peroxisome proliferator-activated receptor γ coactivator 1α mRNA expression, a key regulator of mitochondrial biogenesis. (D) Representative Coomassie-stained blue-native polyacrylamide gel electrophoresis (BN-PAGE) showing the assembly of ETC complexes and supercomplexes (left), with densitometric quantification (right). Data were normalized to a total level of ETC supercomplexes and complexes and expressed as a fold change compared to the WT group. (E) Enzymatic activity of individual ETC complexes assessed using commercial assay kits. *n* = 6 per group. Data are represented as means ± SEM; *P < 0.05, **P < 0.01, and ***P < 0.001. ns, not significant. Data were analyzed via 2-way ANOVA, followed by the Bonferroni post hoc test.

To investigate the mechanisms underlying Mtfr1l deficiency exacerbated mitochondrial dysfunction, we examined the expression of ETC complex subunits using a total OXPHOS antibody cocktail. Western blot analysis revealed minimal changes across groups, with only a modest reduction in complex IV (C IV) observed in WT cardiomyocytes following I/R treatment (Fig. 4A), Consistent with these findings, real-time quantitative polymerase chain reaction (RT-qPCR) analysis showed no significant alterations in mRNA levels of other ETC subunits (Fig. 4B) or peroxisome proliferator-activated receptor γ coactivator 1α (Fig. 4C), the master regulator of mitochondrial biogenesis and energetics.

However, we observed significant alterations in the architecture of ETC complexes and supercomplexes by blue-native polyacrylamide gel electrophoresis (BN-PAGE) analysis. As shown in Fig. 4D, both the total protein levels of individual ETC complexes and the assembly of all supercomplexes were substantially reduced in Mtfr1l-KO mitochondria, under both baseline and I/R conditions. To further assess functional consequences, we measured the enzymatic activities of each ETC complex. All showed reduced activity in Mtfr1l-deficient mitochondria even under sham conditions, with I/R treatment further aggravating these impairments (Fig. 4E). Taken together, these results suggest that MTFR1L does not primarily regulate ETC subunit gene expression but instead plays a pivotal role in facilitating the proper assembly and stabilization of ETC complexes and supercomplexes, an essential requirement for preserving their enzymatic activity and sustaining mitochondrial respiratory function.

### MTFR1L interacts with AIF to maintain its mitochondrial location

**Fig. 5.**
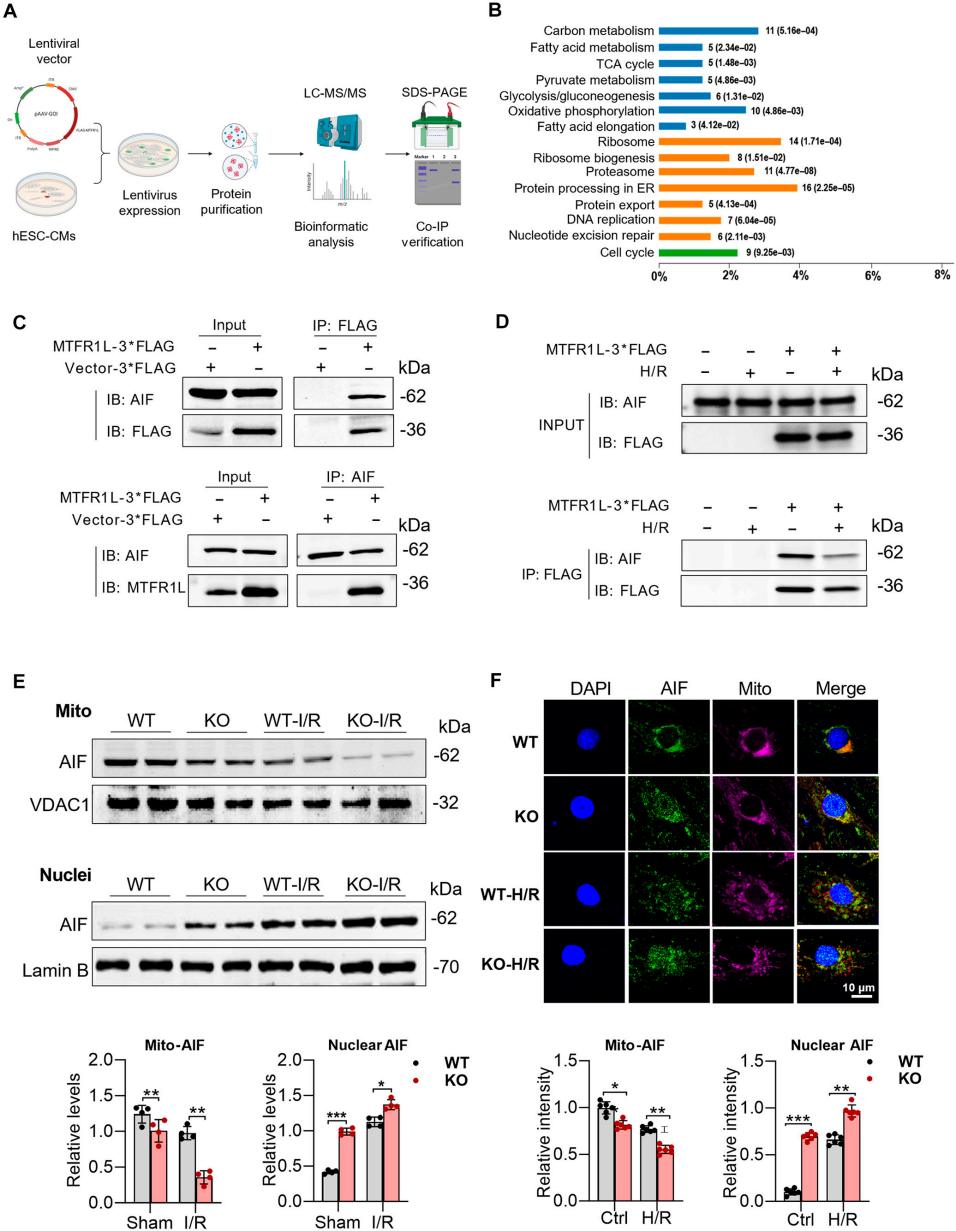
Mitochondrial fission regulator 1-like protein (MTFR1L) interacts with apoptosis-inducing factor (AIF) to restrain apoptosis activation. (A) Schematic overview of the immunoprecipitation (IP)–mass spectrometry workflow used to identify and validate MTFR1L-interacting proteins. *m*/*z*, mass/charge ratio. ITR, inverted terminal repeat; CMV, cytomegalovirus; Ori, origin of replication; pAAV-GOI, adeno-associated virus plasmid carrying gene of interest (GOI, gene of interest); WPRE, Woodchuck hepatitis virus posttranscriptional regulatory element; polyA, polyadenylation signal. (B) Kyoto Encyclopedia of Genes and Genomes pathway enrichment analysis of proteins coprecipitated with MTFR1L. ER, endoplasmic reticulum. (C) Co-IP analysis showing interaction between MTFR1L and AIF in 293T cells transfected with Flag-MTFR1L. Cell lysates were immunoprecipitated with anti-Flag or anti-AIF, followed by immunoblotting (IB) with anti-AIF or anti-Flag antibodies. *n* = 4 per group. (D) Co-IP analysis showing that the interaction between MTFR1L and AIF is reduced in H9 cell-derived cardiomyocytes (H9-CMs) under hypoxia/reoxygenation (H/R) conditions. (E) Representative Western blot showing AIF levels in mitochondrial (top) and nuclear (middle) fractions of wild-type (WT) and Mtfr1l-knockout (KO) mouse hearts under sham or ischemia/reperfusion (I/R) conditions. Quantification is shown at the bottom. (F) Immunofluorescence images of AIF subcellular localization in MTFR1L-KO and WT H9-CMs under sham or H/R. Scale bar, 10 μm. Quantification is shown at the bottom. *n* = 6 per group. Data are represented as means ± SEM; *P < 0.05, **P < 0.01, and ***P < 0.001. Data presented in (E) and (F) were analyzed via 2-way ANOVA, followed by the Bonferroni post hoc test.

Mitochondrial proteins form a highly interconnected and dynamic regulatory network [11,18]. To elucidate MTFR1L’s role within this network and its potential impact on mitochondrial respiratory function, we conducted immunoprecipitation of MTFR1L in H9-CMs, followed by mass spectrometry analysis (Fig. 5A). Among the top 20 identified interactors, we primarily focused on mitochondrial-localized proteins and identified 3 candidates with the highest mitochondrial localization prediction scores (Table S1). Subsequent Kyoto Encyclopedia of Genes and Genomes pathway enrichment analysis revealed that MTFR1L-interacting proteins were significantly enriched in key mitochondrial metabolic pathways, including the citrate (tricarboxylic acid [TCA]) cycle, OXPHOS, and pyruvate metabolism (Fig. 5B). Among the top 3 candidate interactors, AIF stood out because of its well-documented roles in ETC complex assembly, OXPHOS, and caspase-independent apoptosis [19–23]. These functional links prompted us to focus on AIF in subsequent mechanistic studies.

To validate the interaction between MTFR1L and AIF, we transfected human embryonic kidney 293T cells with Flag-tagged MTFR1L and performed coimmunoprecipitation (Co-IP) assays. Reciprocal Co-IP experiments confirmed a specific interaction: AIF was detected in Flag-MTFR1L immunoprecipitates, and, conversely, MTFR1L was also identified in AIF immunoprecipitates (Fig. 5C). Moreover, molecular docking analyses predicted a high-affinity binding between MTFR1L and AIF, as evidenced by favorable docking scores and confidence metrics (Fig. S6A to C). This physical association was further supported by immunofluorescence staining in H9-CMs, which revealed strong colocalization of MTFR1L and AIF (Fig. S6D). To determine whether I/R injury dynamically regulates the MTFR1L–AIF interaction, we subjected WT-H9-CMs to H/R stimulation and performed Co-IP. The amount of AIF detected in MTFR1L immunocomplexes was significantly reduced, indicating that the interaction is dynamically dissociated upon ischemic stress (Fig. 5D).

As AIF mitochondria–nucleus translocation has been implicated in ETC and OXPHOS deficiency, we investigated this event in the context of MTFR1L deficiency and I/R. Both Western blot and immunofluorescence staining analyses revealed a significant reduction in mitochondrial AIF levels in MTFR1L-deficient cardiomyocytes at physiologic conditions, with this deficiency further exacerbated following cardiac ischemia. In contrast, nuclear AIF levels were markedly elevated in MTFR1L-KO cardiomyocytes compared to WT cells, both at baseline and after I/R or H/R treatment (Fig. 5E and F). In addition, the total abundance of AIF protein remained unchanged (Fig. S6E), suggesting that MTFR1L primarily regulates the subcellular localization of AIF rather than its overall expression.

### MTFR1L stabilizes AIF dimers to maintain the CHCHD4–MICOS axis: Safeguarding cristae architecture

**Fig. 6.**
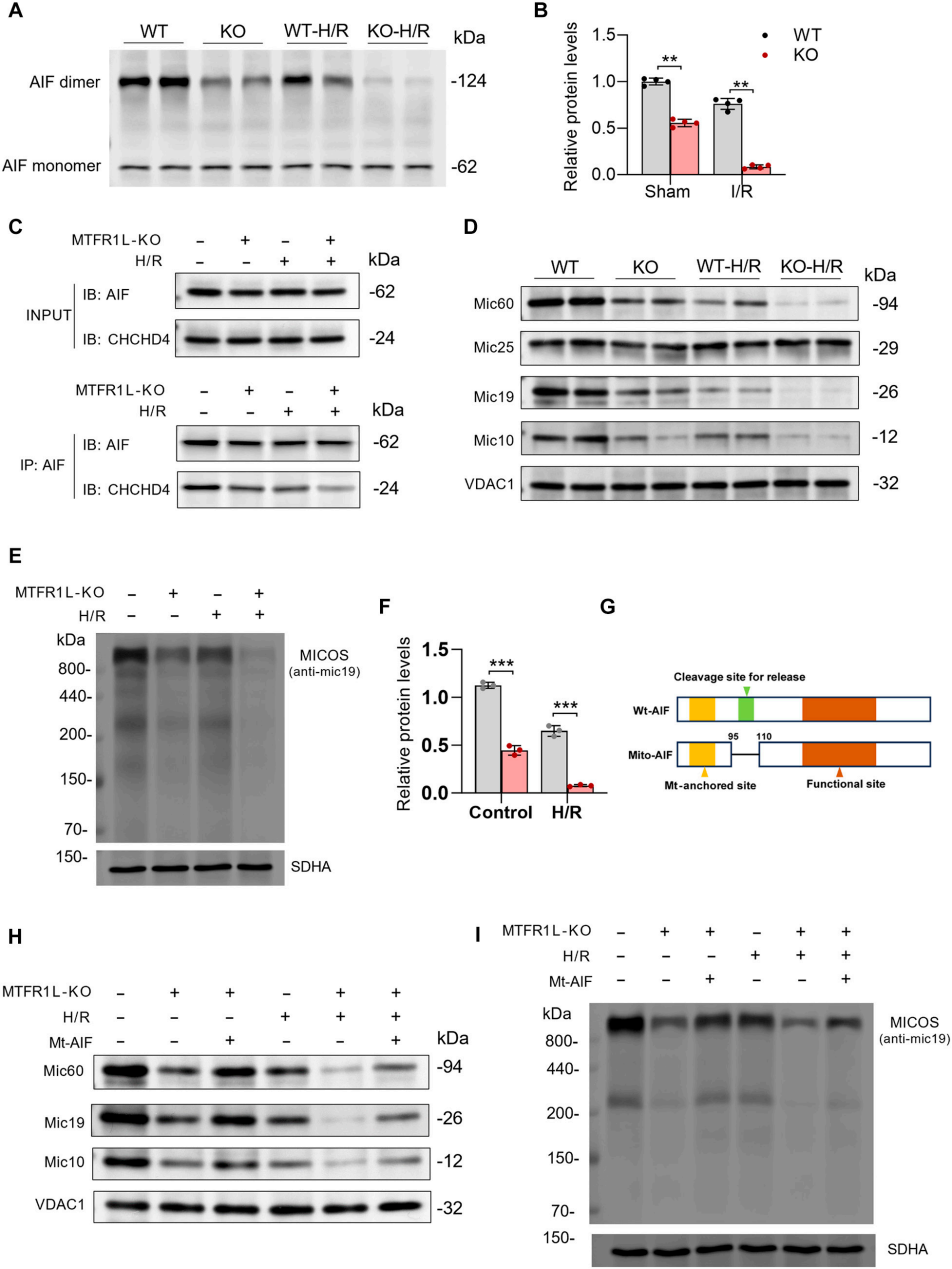
Mitochondrial fission regulator 1-like protein (MTFR1L) stabilizes apoptosis-inducing factor (AIF) dimers to maintain the coiled-coil-helix-coiled-coil-helix domain containing 4 (CHCHD4)–mitochondrial contact site and cristae organizing system (MICOS) axis: safeguarding cristae architecture. (A) Representative Western blot of the levels of AIF dimer in H9 cell-derived cardiomyocytes (H9-CMs) after dextran sulfate sodium-mediated cross-linking. The level of AIF monomers was used as a loading control. (B) Quantification analysis of the AIF dimer/AIF monomer. *n* = 4 per group. (C) Coimmunoprecipitation (Co-IP) analysis of interaction between AIF and CHCHD4 in Mtfr1l-knockout (KO) H9-CMs under basal and hypoxia/reoxygenation (H/R) conditions. (D) Representative Western blot of the levels of MICOS subunits in H9-CMs. Quantification is shown in Fig. S7A. (E) Mitochondrial protein samples were separated by blue-native polyacrylamide gel electrophoresis (BN-PAGE), and MICOS complex was analyzed by Western blot analysis with MIC19 antibody, succinate dehydrogenase complex flavoprotein subunit A (SDHA) served as loading control. (F) Quantification of MICOS complex levels. (G) Schematic illustration of the mitochondria-anchored AIF (Mt-AIF) construct, in which a truncated AIF (lacking amnio acid 96 to 110) is fused with a Flag tag. (H) Representative Western blot of the levels of MICOS subunits in H9-CMs after Mt-AIF overexpression under basal and H/R conditions. Quantification is shown in Fig. S7B. (I) Mitochondrial protein samples were separated by BN-PAGE, and MICOS complex was analyzed by Western blot analysis with MIC19 antibody after Mt-AIF overexpression. SDHA served as loading control. Quantification is shown in Fig. S7C. Data are represented as means ± SEM; *P < 0.05, **P < 0.01, and ***P < 0.001. Data presented in (B) and (F) were analyzed via 2-way ANOVA, followed by the Bonferroni post hoc test.

Mitochondrial AIF exists in a monomer–dimer equilibrium, with dimeric AIF complexed with coiled-coil-helix-coiled-coil-helix domain containing 4 (CHCHD4) to enable the import of mitochondrial contact site and cristae organizing system (MICOS) subunits into the intermembrane space, thereby promoting MICOS assembly and sustaining cristae integrity [24–27]. We therefore investigated whether MTFR1L stabilizes AIF dimers to preserve this axis, defining the molecular link between this interaction and mitochondrial ultrastructure. Cross-linking assays showed that the content of AIF dimers was significantly reduced in H9-MTFR1L-KO-CMs compared to WT under both baseline and H/R conditions, indicating that MTFR1L is essential for maintaining the AIF dimer (Fig. 6A and B). This dimer disruption further impaired AIF–CHCHD4 interaction, as demonstrated by Co-IP showing a significant reduction in CHCHD4 recovery with AIF under both conditions (Fig. 6C). Consequently, import of Mic19, a CHCHD4 substrate and core MICOS subunit required for complex assembly, was compromised. Western Blot revealed markedly reduced mitochondrial Mic19 in H9-MTFR1L-KO-CMs with concomitant destabilization of other MICOS complex subunits, Mic10 and Mic60 (Fig. 6D and Fig. S7A) . Next, to assess the integrity of the MICOS complex, mitochondria from WT and KO H9-CMs under baseline and H/R conditions were separate by BN-PAGE and then analyzed by Western blot analysis. Importantly, MTFR1L-KO results in remarkable reduced MICOS complex content (Fig. 6E and F), indicating pronounced disassembly of the MICOS, which paralleled the severe cristae abnormality observed by electron microscopy above.

To further verify that AIF functions downstream of MTFR1L to regulate mitochondrial architecture, we overexpressed Mt-AIF (Fig. 6G) in H9-MTFR1L-KO-CMs and examined whether this intervention could rescue MICOS integrity and cristae morphology. Under both baseline and H/R conditions, Mt-AIF restored Mic19 import (Fig. 6H and Fig. S7B). Consequently, levels of associated MICOS subunits Mic10 and Mic 60 also rose markedly (Fig. 6H and Fig. S7B), and BN-PAGE showed robust reassembly of the MICOS complex (Fig. 6I and Fig. S7C). Transmission electron microscopy further confirmed these findings (Fig. S8A). Mt-AIF-expressing MTFR1L-KO cardiomyocytes exhibited pronounced restoration of cristae architecture, with more regularly aligned cristae, an increased number of well-defined cristae junctions, and shortened cristae lumen (Fig. S8B to E). Importantly, despite improving cristae architecture, Mt-AIF did not reverse the mitochondrial hyperfusion phenotype, nor did it alter the expression of canonical dynamics regulators including DRP1, MFN1/2, and OPA1 (Fig. S9), confirming that structural rescue is independent of canonical dynamics modulation. Together, these findings establish that MTFR1L regulates mitochondrial cristae morphology exclusively via an AIF-dependent axis—stabilizing AIF dimers to enable AIF/CHCHD4-mediated MICOS assembly, thereby providing the molecular mechanism by which MTFR1L safeguards mitochondrial ultrastructure against cardiac I/R injury.

### Mt-AIF overexpression improves MTFR1L-deficiency-exacerbated I/R injury

**Fig. 7.**
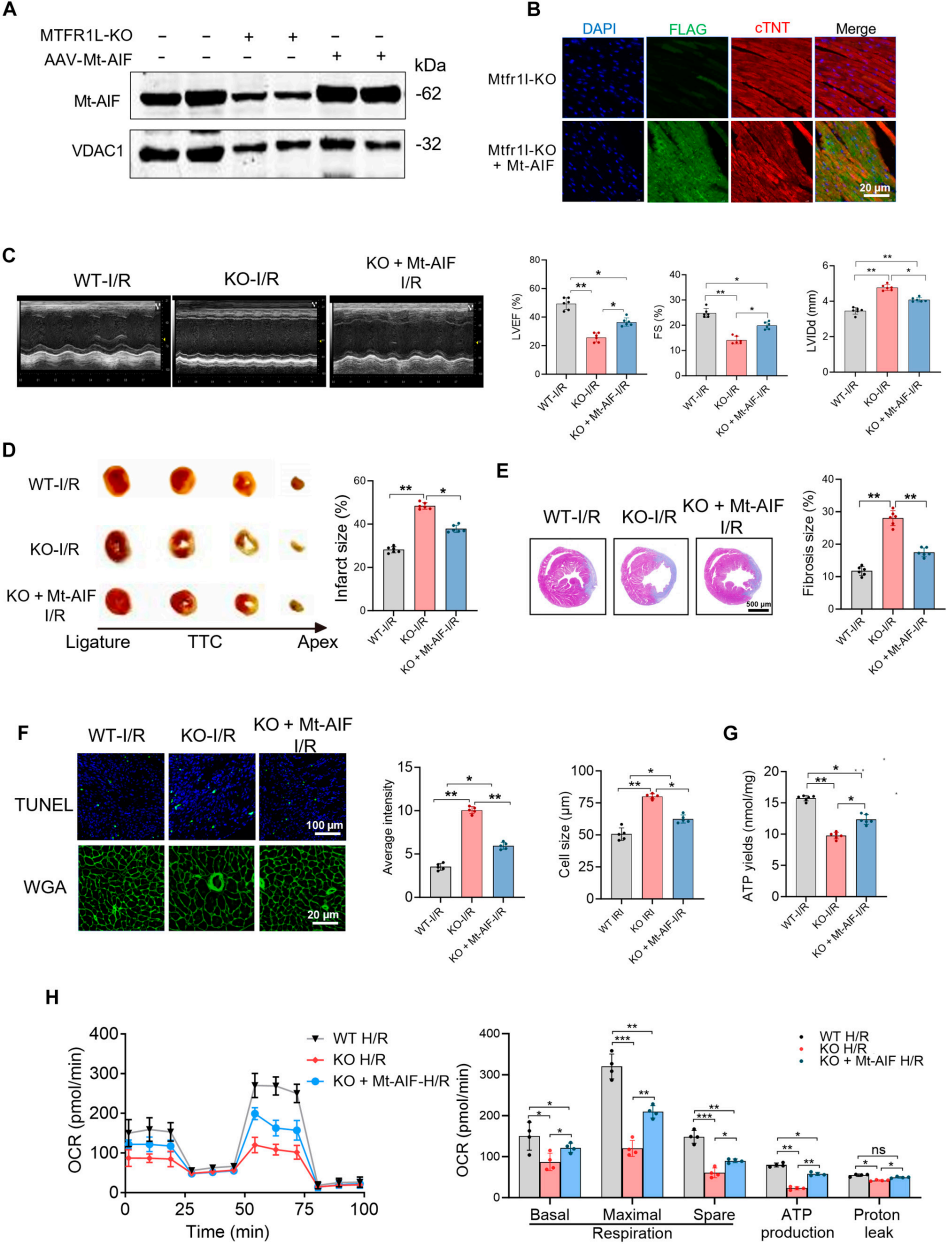
Mitochondria-anchored apoptosis-inducing factor (Mt-AIF) restores cardiac function in mitochondrial fission regulator 1-like protein (Mtfr1l)-knockout (KO) mice following ischemia/reperfusion (I/R) injury. (A) Western blot analysis of mitochondrial AIF levels in Mtfr1l-KO mouse hearts following a single intravenous injection of adeno-associated virus 9 (AAV9)-Flag-Mt-AIF. (B) Immunofluorescence staining of cardiac troponin T (cTnT; red) and Flag (green) confirming myocardial overexpression of Mt-AIF in Mtfr1l-KO mice. Scale bar, 20 μm. (C) Representative echocardiograms and quantitative analysis of cardiac function in wild-type (WT), Mtfr1l-KO, and Mtfr1l-KO + Mt-AIF groups under I/R conditions. (D) TTC (2,3,5-triphenyltetrazolium chloride) staining and quantification of infarct size. *n* = 6 per group. (E) Masson trichrome staining of myocardial fibrosis and quantification. Scale bar, 500 μm. (F) Terminal deoxynucleotidyl-transferase-mediated deoxyuridine triphosphate nick end labeling (TUNEL) and wheat germ agglutinin (WGA) staining to assess apoptosis and cardiomyocyte size, respectively, with quantification. Scale bars, 100 and 20 μm. (G) Adenosine triphosphate (ATP) levels were measured under I/R conditions to assess mitochondrial bioenergetic function across groups. (H) Seahorse Mito Stress Test evaluating the rescue effect of Mt-AIF under hypoxia/reoxygenation (H/R) treatment. Left: Real-time oxygen consumption rate (OCR) profiles. Right: Quantification of mitochondrial respiration parameters. Data are represented as means ± SEM; *P < 0.05, **P < 0.01, and ***P < 0.001. ns, not significant. Data presented in (C) to (H) were analyzed via one-way ANOVA, followed by the Bonferroni post hoc test.

To investigate the role of AIF in MTFR1L-mediated cardioprotection against cardiac I/R injury, we used a gain-of-function approach in Mtfr1l-KO mice. Specifically, we designed an adeno-associated virus 9 (AAV9) vector encoding a​ FLAG-tagged Mt-AIF construct to prevent AIF release from mitochondria during stress conditions [28]. Mtfr1l-KO mice received a single intravenous injection of AAV9-Mt-AIF 4 weeks prior to I/R surgery. High expression of mitochondrial anchored AIF expression was detected in cardiomyocytes within 21 d postinjection (Fig. 7A and B).

Cardiac function study revealed that Mt-AIF overexpression significantly alleviated I/R-induced cardiac dysfunction, as indicated by improved LVEF, FS, and reduced LVIDd (Fig. 7C), along with lower serum levels of CK-MB and LDH (Fig. S10A and B). Moreover, Mt-AIF overexpression attenuated structural remodeling in Mtfr1l-KO hearts post-I/R, with reduced infarct size and fibrosis area (Fig. 7D and E). Consistently, Mtfr1l-KO mice expressing Mt-AIF exhibited suppressed cardiomyocyte apoptosis and hypertrophy (Fig. 7F), accompanied by enhanced ATP production (Fig. 7G).

Next, we infected H9-CMs with lenti-Mt-AIF and assessed its mitochondrial localization. To verify that exogenous Mt-AIF is properly anchored, we treated WT cells with actinomycin D + Z-Val-Ala-Asp-fluoromethylketone (zVAD-fmk) to induce endogenous AIF release; Mt-AIF remained localized to mitochondria and effectively blocked nuclear translocation (Fig. S10C). In MTFR1L-KO cells subjected to H/R stress, Mt-AIF attenuated H/R-induced injury, evidenced by reduced LDH release and increased cell viability (Fig. S10D and E). Seahorse analysis further revealed that Mt-AIF overexpression restored mitochondrial respiration impairment in H9-MTFR1L-KO-CMs under H/R treatment across all key parameters (Fig. 7H). In addition, Mt-AIF infection suppressed oxidative stress, as indicated by reduced level of ROS, MDA, and 4-HNE production (Fig. S10F to H).

### Postischemic overexpression of Mt-AIF has therapeutic effects against cardiac I/R injury

**Fig. 8.**
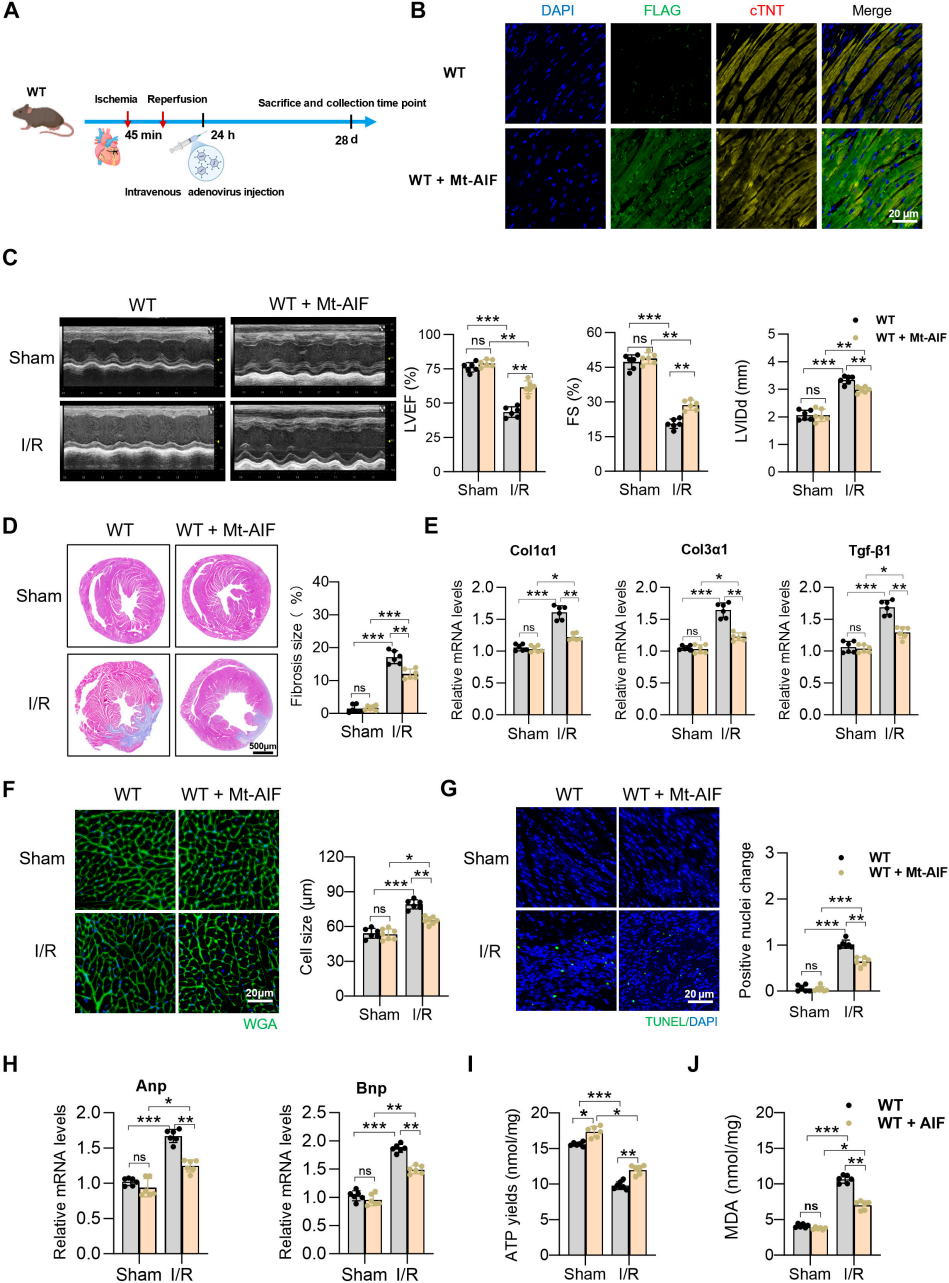
Postischemic overexpression of mitochondria-anchored apoptosis-inducing factor (Mt-AIF) has therapeutic effects against cardiac ischemia/reperfusion (I/R) injury. (A) Schematic representation demonstrating that mice received adeno-associated virus 9 (AAV9)-FLAG-Mt-AIF treatment within 24 h after myocardial reperfusion. Heart tissues were collected 4 weeks after cardiac I/R injury. (B) Immunofluorescence staining of cardiac troponin T (cTnT; golden) and Flag (green) confirming myocardial overexpression of Mt-AIF in wild-type (WT) mice. Scale bar, 20 μm. (C) Representative echocardiograms and quantitative analysis of cardiac function in mice 4 weeks after cardiac I/R injury. *n* = 6 per group. (D) Masson trichrome staining of myocardial fibrosis and quantification. Scale bar, 500 μm. (E) Real-time quantitative polymerase chain reaction (RT-qPCR) analysis of fibrosis marker genes expression 28 d after I/R. *n* = 6 per group. (F) Wheat germ agglutinin (WGA) staining to cardiomyocyte size with quantification. *n* = 6 per group. Scale bar, 20 μm. (G) Terminal deoxynucleotidyl-transferase-mediated deoxyuridine triphosphate nick end labeling (TUNEL) staining and analysis of apoptosis level in mice heart 28 d after I/R. Scale bar, 100 μm. (H) RT-qPCR analysis of cardiac remodeling marker genes 28 d after I/R. *n* = 6 per group. (I) Adenosine triphosphate (ATP) levels were measured 28 d after I/R to assess mitochondrial bioenergetic function across groups. *n* = 6 per group. (J) Quantification of oxidative stress markers malondialdehyde (MDA) in heart tissues by immunoassay 28 d after I/R. *n* =6 per group. Data are represented as means ± SEM; *P < 0.05, **P < 0.01, and ***P < 0.001. ns, not significant. Data presented in (C) to (J) were analyzed via 2-way ANOVA, followed by the Bonferroni post hoc test.

Given that therapeutic interventions are typically administered after the onset of cardiac I/R injury in clinical practice, we further explored the therapeutic potential of Mt-AIF overexpression (Fig. 8A). WT mice were received intravenous injection of AAV9-FLAG-Mt-AIF within 24 h after myocardial reperfusion. Overexpression of Mt-AIF in heart tissues was validated 4 weeks after I/R injury (Fig. 8B). After that, we demonstrated that the AAV9-FLAG-Mt-AIF treatment effectively preserved cardiac function and attenuated cardiac remodeling (Fig. 8C to H) . Mice treated with AAV9-FLAG-Mt-AIF also exhibited improved mitochondrial function, as indicated by increased ATP levels and less oxidative stress levels (MDA levels) in heart tissues (Fig. 8I and J). Collectively, these data suggest that Mt-AIF overexpression remains an effective therapeutic approach for protection against cardiac I/R injury.

## Discussion

Numerous studies have revealed that mitochondria are multifaceted organelles, serving as central hubs for a wide range of cellular functions. Accordingly, maintaining mitochondrial homeostasis is crucial for sustaining cell survival and enabling appropriate responses to various environmental cues. Among the key mechanisms regulating mitochondrial homeostasis, mitochondrial dynamics, specifically referred as fusion and fission, have been extensively studied over the past decades [29]. Targeting mitochondrial dynamics proteins that are dysregulated in disease has been represent a potential therapeutic strategy for various diseases [5,30,31].

As a newly identified mitochondrial dynamics protein, MTFR1L is highly expressed in cardiomyocytes, which are densely packed with mitochondria to meet the heart’s high energy demands, suggesting that MTFR1L plays an important role in the pathogenesis of cardiovascular disease. In this study, we first examined its expression in a mouse model of I/R injury and observed a distinctive expression pattern: a rapid decline in MTFR1L levels within 1 d of I/R treatment, followed by a gradual return over time. Based on previous studies demonstrating that MTFR1L deficiency promotes mitochondrial fusion [12], we suppose that cardiomyocytes initially favor mitochondrial fusion as an acute, adaptive response to I/R-induced mitochondrial damage. This fusion process facilitates functional complementation by mixing mitochondrial contents and mitochondrial DNA, thereby offering a relatively low-cost compensatory mechanism to preserve mitochondrial function. However, this initial fusion response is likely insufficient to counteract the severity and persistence of I/R injury, which is possibly sensed by yet-unknown mechanisms that subsequently restore MTFR1L expression to near-baseline levels to further address the I/R injury.

Consistent with previous study [12], our investigation into the consequences of MTFR1L loss of function also revealed increased mitochondrial fusion events in MTFR1L-deficient cardiomyocytes, as evidenced by a marked increase in mitochondrial area and a reduction in mitochondrial number observed from electron microscopic images. Mechanistically, this fusion phenotype was attributed to elevated OPA1 protein levels, consistent with MTFR1L deficiency up-regulating OPA1 to drive mitochondrial membrane fusion [12]. Unexpectedly, these mitochondrial morphologic alterations had minimal impact on animal survival, cardiac function, and cardiac remodeling under basal conditions. This may be attributed to the only mild ROS accumulation and a slight reduction in mitochondrial potential caused by loss of MTFR1L. Furthermore, although our findings showed that loss of MTFR1L leads to impaired ETC respiration capacity due to disassembly of ETC supercomplexes, the slightly declined cardiac function observed in Mtfr1l-KO mouse models implies that this level of mitochondrial dysfunction and structural abnormalities is still sufficient to partially meet the heart’s baseline energy demands and maintain basic mitochondrial homeostasis under physiological conditions.

In contrast, under pathological conditions, here in the setting of I/R injury, mitochondrial defects resulting from MTFR1L-KO failed to counteract the intense cellular stress and instead significantly exacerbated I/R-induced cardiac damage. This was evidenced by increased mortality, more pronounced cardiac dysfunction, and aggravated cardiac remodeling in Mtfr1l-KO mice compared to WT controls. Further investigation revealed that I/R treatment caused pronounced disruptions to mitochondrial cristae in Mtfr1l-KO cardiomyocytes, which exhibited a substantial reduction in cristae number and junctions, along with a notable widening of the cristae lumen.

Mitochondrial cristae are lamellar invaginations of the inner mitochondrial membrane, where ETC complexes and their higher-order assemblies, ETC supercomplexes, are precisely organized [32]. ETC supercomplexes have been well characterized to serve to stabilize individual respiratory complexes, facilitate efficient electron transfer, and limit excessive ROS production [33]. Our study showed that in I/R-treated MTFR1L-deficient cardiomyocytes, the extensive cristae disorganization resulted in severe disassembly of ETC supercomplexes and markedly compromised enzymatic activity of individual ETC complexes. These defects directly lead to profound mitochondrial dysfunction, including markedly reduced ATP production, severely impaired mitochondrial respiration, substantial ROS and oxidative by-product accumulation, and a significant decline in ΔΨm, which collectively underlie the exacerbated cardiac injury caused by I/R stress.

While our study confirms some of the findings of previous research, the results differ in some ways from previous study on the role of MTFR1L in mitochondria [12]. The recent study observed a decrease in cristae width and also inferred that loss of MTFR1L, mimicking mild cellular stress, induces a metabolic adaption based on the result of decreased activation of AMPK with no change in the ratio of ATP/adenosine diphosphate [12]. Nevertheless, our results showed that depletion of MTFR1L widens the cristae lumen, impairs mitochondrial respiratory function, and increases oxidative stress in vivo and in hESC-CMs, thus exacerbating cardiac I/R injury. The difference between recent study and ours may be attributed to variations in research models, experimental design, and methodologies in detection. The observed differences in mitochondrial cristae may stem from the intrinsic structural differences of mitochondria due to cell type and tissue characteristics. In the recent study, U2OS cells, which are often in a state of rapid proliferation, may have relatively immature mitochondrial structures with sparser cristae as observed under electron microscopy. In contrast, the subject of our study, mouse cardiac tissue, is a highly differentiated organ, and to meet the demand for efficient energy metabolism, its mitochondria are more developed with abundant cristae structures and, thus, may be more representative. In addition, in our research, we studied the function of MTFR1L in the heart and cardiomyocytes, where it is highly expressed, indicating its important role in regulating mitochondrial quality, so loss of MTFR1L may lead to mitochondrial dysfunction. By contrast, previous study used U2OS cell lines in which the function of MTFR1L may not be dominant. In addition, mitochondrial function in the U2OS cell was detected under glucose starvation, which is a mild stress model; however, in our study, it was detected under a much more severe condition of hypoxia. Conceivably, MTFR1L may play a much more important role in the heart and under extreme stress conditions.

In fact, it has been well established that mitochondrial dynamics, cristae architecture, and respiratory function are tightly interconnected. Mitochondrial fusion and fission not only govern the morphological plasticity of the network but also influence the structural organization of cristae, which are essential for optimal assembly and function of the ETC. Notably, fusion-related proteins such as OPA1 play dual roles in both membrane tethering and cristae maintenance, thereby directly linking dynamics to bioenergetic capacity [34]. Disruption of cristae structure often leads to impaired respiratory efficiency and ATP production, while defects in OXPHOS can, in turn, activate fission and mitophagy pathways as compensatory responses [35]. In our study, the findings imply that the fission-related protein MTFR1L works as a key coordinator among mitochondrial dynamics, cristae remodeling, and respiratory activity to sustain mitochondrial homeostasis and cellular energy metabolism and to ultimately exert a cardioprotective effect during I/R injury.

To elucidate the molecular basis of this coordination, we investigated how MTFR1L maintains cristae architecture. Through immunoprecipitation–mass spectrometry screening, we identified a physical interaction between MTFR1L and AIF, a flavoprotein localized to the mitochondrial intermembrane space that moonlights as a regulator of both mitochondrial function and cell death [36,37]. Under physiological conditions, AIF exists in a monomer–dimer equilibrium, with the dimeric form serving as a scaffold for CHCHD4 to facilitate the import different mitochondrial protein subunits required for cristae organization and ETC complex assembly into the inner mitochondrial membrane [24,25,38,39]. Our Co-IP experiment confirmed a physical interaction between MTFR1L and AIF, suggesting that MTFR1L directly binds to and retains AIF within mitochondria. Mechanistically, MTFR1L deficiency led to AIF dimer dissociation, thereby disrupting AIF/CHCHD4-dependent MICOS assembly and resulting in cristae junction loss, lumen widening, and ETC supercomplex disassembly. Rescue experiments further demonstrated that Mt-AIF overexpression simultaneously restored MICOS assembly and partially recovered cristae architecture, confirming AIF as the critical downstream effector of MTFR1L. Notably, Mt-AIF overexpression did not alter the expression or phosphorylation status of mitochondrial dynamics regulators (DRP1, MFN1/2, and OPA1) under either basal or H/R conditions. At the structural level, studies show that the AIF homodimer adopts a “head-to-head” symmetric configuration mediated by the flavin adenine dinucleotide-binding domain (amino acids 120 to 270), with the dimer interface stabilized by salt bridges [27]. In the present study, molecular docking analysis predicted that MTFR1L binds to specific sites on the AIF monomer (including Thr-252, Ser-209, Lys-112, and Ser-76), with high affinity (Δ*G* = −44.97 kcal/mol). Notably, these binding sites are located at the periphery of the flavin adenine dinucleotide-binding domain, spatially positioned on the outer surface of the AIF homodimer without overlapping the dimer interface. This suggests that MTFR1L potentially serves as a “molecular clamp” that stabilizes the oxidized, dimeric conformation by occupying peripheral pockets adjacent to the dimer interface, thereby preventing monomer dissociation. These structural insights provide a mechanistic basis for MTFR1L-mediated AIF retention and dimer stabilization, maintaining the scaffold necessary for CHCHD4 recruitment.

In addition to its role in preserving mitochondrial ultrastructure, this study reveals a previously unrecognized cardioprotective contribution of MTFR1L in regulating mitochondria-associated apoptosis, a critical driver of myocardial injury during I/R stress [40]. Upon apoptotic stimuli, such as I/R injury, AIF is released from mitochondria and subsequently translocates to the nucleus, where it induces chromatin condensation and large-scale DNA fragmentation, ultimately leading to caspase-independent apoptosis [20,41]. Our results showed that the MTFR1L–AIF interaction was significantly attenuated under H/R stress, accompanied by decreased mitochondrial AIF, increased nuclear accumulation, and enhanced cardiomyocyte apoptosis. Cardiac-specific overexpression of Mt-AIF significantly attenuated the exaggerated myocardial infarct size and improved cardiac function in MTFR1L-deficient mice following I/R injury. Notably, these results confirm that structural and apoptotic functions are unified through the MTFR1L–AIF axis: By ensuring mitochondrial retention of AIF, MTFR1L simultaneously preserves cristae integrity and prevents nuclear translocation, thereby exerting dual cardioprotective effects. These findings have direct translational relevance for myocardial I/R injury, where current reperfusion therapies lack effective pharmacological protection. Stabilizing the MTFR1L–AIF axis—either by enhancing their interaction or delivering Mt-AIF—offers a dual-target strategy preserving both mitochondrial structure and cell viability, with potential as an adjunctive therapy alongside standard PCI or thrombolysis.

Taken together, all the findings of this study reveal the cardioprotective function of MTFR1L and elucidate its underlying mechanisms from 2 perspectives: On one hand, it possibly works with AIF to maintain mitochondrial cristae integrity, ETC complexes, and supercomplex assembly; on the other hand, it tethers AIF within mitochondria to prevent its release and the subsequent activation of AIF-mediated apoptosis. Our research provides new insights into the role of MTFR1L and highlights it as a promising therapeutic target for myocardial I/R injury.

## Materials and Methods

### Cell lines, culture, cardiac differentiation, and treatment

A WT hESC line, H9 cells (WA09, WiCell, RRID:CVCL_9773), was obtained from WiCell Research Institute (Madison, WI, USA). The cell line was authenticated by short tandem repeat profiling and confirmed to be free of contamination. An MTFR1L-KO hESC line was generated from the H9 line via Cas9-mediated frameshift mutation. Briefly, we designed an sgRNA (sequence: 5′-CAGGAGCTGGTTCAAATGTC-3′) targeting exon 2 of the MTFR1L gene. This sgRNA sequence was then cloned into a Cas9-expressing episomal vector (PX458-T2, Addgene, #113194). The constructed plasmids were subsequently electroporated into H9 cells (Lonza Bioscience, Germany). Three days postelectroporation, transfected cells were sorted by green fluorescent protein using fluorescence-activated cell sorting and dispensed into a 96-well plate. After approximately 10 d of culturing, each cell clone was passaged and expanded for DNA extraction, followed by PCR and Sanger sequencing. Clones with nontriple frameshift mutations were selected for line construction, and Western blot was performed to validate MTFR1L expression, ultimately establishing the H9-MTFR1L-KO line. To test for off-target mutations, we used CCTop online predictor (https://cctop.cos.uni-heidelberg.de) to search potential off-target sites [42]. The top 5 candidate regions within gene exons were PCR-amplified and subjected to Sanger sequencing (Fig. S3).

All hESCs were cultured in feeder-free plates precoated with Geltrex (Life Technologies, USA) and maintained in PSCeasy medium (Cellapy, China). Once cells reached 80% to 90% confluence, they were induced to differentiate into cardiomyocytes using chemically specific small molecules that modulate Wnt/β-catenin pathway [43] with minor modifications (Fig. S1B). Briefly, cells were initially treated with basic medium (RPMI 1640 plus 1× B27 minus insulin) containing 6 μM CHIR99021 (STEMCELL Technologies, Canada) for 48 h. This was followed by basic medium containing 2 μM IWR-1-endo (STEMCELL Technologies, Canada) for another 48 h. After 6 more days cultured in basic medium, cardiomyocytes were enriched by culturing in lactate-containing medium for less than 3 d [44]. Cells were then recovered for at least 3 d in RPMI 1640 medium. Unless otherwise indicated, differentiated cardiomyocytes were used between days 30 and 40 of differentiation.

For H/R experiments, cardiomyocytes were first treated with normal or glucose-free medium overnight (18 h) in either normoxic (~21% O_2_ and 5% CO_2_) or hypoxic (1% O_2_ and 5% CO_2_) cell culture incubators. Subsequently, the cells were changed back to normal medium and incubated under standard incubation conditions for another 2 h.

Human embryonic kidney 293T cells (catalog no. CRL-3216, American Type Culture Collection, RRID: CVCL_0063) were cultured in complete culture medium (Dulbecco’s modified Eagle’s medium supplemented with 10% fetal bovine serum and 1% penicillin–streptomycin) and passaged using Trypsin for cell dissociation.

### Animal experiments

Mtfr1l-KO mice were generated by Shanghai Model Organisms Center Inc. and then backcrossed onto a C57BL/6J background. All mice were housed in a suitable barrier system with controlled temperature (23 ± 1 °C) and humidity (45% to 55%), a 12-h light-dark cycle, and free access to water and standard rodent diet. Mice aged 10 to 12 weeks were selected to undergo induce I/R injury induced by left anterior descending artery ligation. Briefly, mice were fully anesthetized with 2% isoflurane, and their thoracic cavity was opened to expose the heart. The left anterior descending artery, 1 mm lower than the tip of the left atrial appendage, was then ligated with a slipknot using a 7-0 silk suture. Ischemia was confirmed by ST segment elevation on electrocardiogram and visual blanching. After 45 min of occlusion, the slipknot was released to achieve reperfusion.

To specifically overexpress Mt-AIF in mouse heart, Mtfr1l-KO mice were given a single intravenous injection of AAV9-FLAG-Mt-AIF at a dose of 1 × 10^11^ viral genomes 4 weeks prior to I/R.

At the end point of the animal experiments, mice were anesthetized with 4% isoflurane inhalation and subsequently euthanized by cervical dislocation. All animal procedures were approved by the Animal Welfare and Ethics Branch, Biomedical Ethics Committee of Peking University and followed the Guide for the Care and Use of Laboratory Animals established by the National Institutes of Health.

### Echocardiogram evaluation

Cardiac contractile function was measured by transthoracic 2-dimensional echocardiography (Vevo 2100, FUJIFILM VisualSonics, Canada). Briefly, mice were anesthetized with 2% isoflurane. Two-dimensional guided M-mode images, perpendicular to the maximum left ventricular dimension, were recorded during end-diastole and systole for at least 5 consecutive cardiac cycles to assess wall thickness and chamber dimensions. LVEF and FS were then calculated from the M-mode data using VevoLAB analysis software (v5.6.1, FUJIFILM VisualSonics, Canada).

### Histological analysis

TTC (2,3,5-triphenyltetrazolium chloride) staining was used to evaluate the mouse cardiac infarct size. Excised heart samples were frozen at −80 °C for 10 min and then sectioned into 1-mm slices using a sectioning mold (catalog no. SQP-X7, Servicebio, China). The slices were subsequently incubated with 12% TTC solution at 37 °C for 20 min. Normal tissue will be stained red, whereas the infarcted part either remains unstained or appears lightly stained. The infarct size was quantified and analyzed by Image-Pro Plus 6.0 software. For Masson’s trichrome and WGA staining, excised heart samples were fixed in 4% paraformaldehyde, dehydrated, then embedded in paraffin, and sliced into 5-μm sections. The cardiac slices were incubated with WGA working solution at 37 °C for 30 min. Masson’s trichrome staining was performed according to the standard protocols [45].

### Immunofluorescence and immunohistochemical staining

For tissue samples, paraffin-embedded hearts were cut into 5-μm slices, then processed to deparaffinization and rehydration, and underwent antigen retrieval. For cell samples, cells were seeded onto coverslips, fixed with 4% paraformaldehyde for 15 min, and permeabilized in 1% Triton X-100 for 7 min at room temperature. Cardiac slices or cell coverslips were then blocked with bovine serum albumin for 1 h at room temperature. Next, cells were incubated with the primary antibodies at 4 °C overnight, followed by incubation with fluorescent secondary antibodies at 37 °C in the dark for 1 h. Immunofluorescent images were captured using a confocal laser scanning microscope (FV3000, Olympus, Japan).

Immunohistochemical staining was performed to examine Mtfr1l expression level across different organs of mice. The paraffin sections underwent deparaffinization, rehydration, and antigen retrieval. Next, tissue slices were incubated with anti-MTFR1L at 4 °C overnight, followed by incubation with horseradish-peroxidase-conjugated secondary antibodies at 37 °C for 1 h. Images were captured using a Leica DMi8 microscope (Leica Microsystems, Germany).

### Transmission electron microscopy

The excised heart was dissected into 1 mm × 1 mm × 1 mm pieces and fixed in cold 2.5% glutaraldehyde. Then, the tissues were dehydrated and embedded in resin. Subsequently, ultrathin sections of 60 to 80 nm were prepared using an ultramicrotome. Images were captured by a transmission electron microscope (HT7700, Hitachi, Japan).

Quantitative analysis of mitochondrial parameters was performed using the National Institutes of Health ImageJ software, following a published universal approach [46]. Briefly, region of interest manager was used to record and keep track of measurements such as area, length, circularity, and perimeter. To ensure accurate and reproducible results, these measurements should be performed on at least 3 samples per group, with a minimum of 20 mitochondria analyzed per sample, distributed across multiple different fields of view.

### TUNEL, ROS, and ΔΨm detection

TUNEL staining was performed to evaluate cell apoptosis levels in mouse hearts using a commercial kit (Beyotime, China), with paraffin-embedded slices prepared according to the manufacturer’s instructions. Apoptotic cells were identified by green fluorescence, and images were captured using a fluorescence microscope. The percentage of TUNEL-positive cells was quantified relative to the total number of nuclei (stained with 4′,6-diamidino-2-phenylindole [DAPI]) in multiple fields.

For ROS detection, fresh frozen mouse hearts were sectioned into 5-μm slices. These were then incubated with prepared dihydroethidium working solution (Servicebio, China) at 37 °C for 30 min in the dark. Dihydroethidium fluorescence intensity, indicating ROS levels, was measured from captured images using image analysis software (e.g., ImageJ) in multiple randomly selected fields.

ΔΨm was measured by the fluorescent probe JC-1 in accordance with the established protocol (Solarbio, China). Briefly, fresh mouse heart tissue was immediately processed into frozen sections, which were incubated with JC-1 (2.5 mg/ml) at room temperature for 20 min in the dark and subsequently washed 3 times with phosphate-buffered saline. Images were acquired with a fluorescence microscope, and ΔΨm was determined by quantifying the ratio of red (JC-1 aggregates) to green (JC-1 monomers) fluorescence intensity using image analysis software in multiple fields.

### Mitochondrial stress test

Cardiomyocytes mitochondrial respiration capacity was evaluated using the mitochondrial stress test (Agilent, USA). Briefly, H9 or H9-MTFR1L-KO-CMs were dissociated and seeded onto a Geltrex-precoated 24-well Seahorse analyzer plate at a density of 4 × 10^4^ to 5 × 10^4^ cells per well and then cultured in RPMI 1640 + B27 medium for 3 d. After H/R treatment, oxygen consumption rate (OCR) was measured using a Seahorses Bioscience XF24 extracellular flux analyzer according to the manufacturer’s manual and normalized to cell number. OCR was expressed as picomoles per minute per 10^3^ counts.

### Animal serum and tissue biochemical analysis

For serum biochemical analysis, fresh mouse blood samples were allowed to clot at room temperature for 2 h, followed by centrifugation at 3,000 rpm for 15 min at 4 °C. The resulting supernatant was collected and immediately used to assess levels of CK, CK-MB, and LDH using an automated biochemical analyzer (Mindray, China).

For tissue analysis, freshly harvested heart tissues were homogenized or lysed to measure the levels of MDA, 4-HNE, and 3-NT using commercial kits. In addition, ATP levels in whole heart tissue and isolated cardiomyocytes were quantified using a commercial ATP assay kit (Beyotime, China).

### ETC complex and supercomplex analyses

Mitochondria were isolated from heart tissues using a commercial kit (Beyotime, China). Briefly, fresh heart tissues were collected and kept on ice. Approximately 50 mg of tissue was cut into small pieces and then incubated in ice-cold trypsin solution on ice for 20 min. Next, the pieces were combined with ice-cold mitochondria isolation reagent and homogenized on ice for 20 to 30 strokes. The homogenate was then centrifuged at 600*g* for 5 min at 4 °C, and the resulting pellet represented the isolated mitochondria.

Then, mitochondrial ETC supercomplexes were analyzed by BN-PAGE. Briefly, mitochondrial membrane proteins were extracted from isolated mitochondria using mitochondria lysis buffer (Beyotime, China) supplemented with 20% digitonin. The protein solution was then mixed with BN-PAGE sample buffer (2×) in a 1:1 ratio. Electrophoresis was conducted using a BN-PAGE gel. After electrophoresis, gels were stained with Coomassie brilliant blue G250 and destained using Coomassie blue destaining solution.

For enzymatic activity measurement, mitochondrial proteins extracted from fresh hearts were incubated with specific substrates for each ETC complex. The change in absorbance at designated wavelengths was recorded (Elabscience, China), and enzymatic activity was subsequently calculated on the basis of standard formulas derived from the rate of absorbance change.

### Co-IP and mass spectrometry

H9-CMs were first infected with lenti-MTFR1L-3×FLAG for 72 h. Then, radioimmunoprecipitation assay lysis buffer supplemented with protease inhibitor, phosphatase inhibitor, and phenylmethylsulfonyl fluoride (1:100) was added to lyse the cells. The collected cell lysis was mixed with anti-Flag antibody or immunoglobulin G and incubated at 4 °C for 6 h. Afterward, Protein A + G agarose beads were added in the mixture and shaken overnight at 4 °C. The antibody–protein complexes were washed and collected for Western blot or mass spectrometry analysis. Mass spectrometry analysis was conducted by Bio-Tech Pack Technology Company Ltd. Briefly, samples were first subjected to reductive alkylation, followed by trypsin digestion. The resulting peptides were then analyzed by liquid chromatography–tandem mass spectrometry (LC-MS/MS) to generate raw data files. Subsequent data processing and protein identification were carried out using MaxQuant software (version 1.6.2.10).

### Lentiviral and AAV9 vector construction

The lentiviral construct pLV-3×FLAG-hMTFR1L, driven by the elongation factor 1α (EF1α) promoter, was designed and produced by VectorBuilder Inc. (China) using NM_001099625.2 as the reference transcript for MTFR1L. AAV9-FLAG-mt-AIF was constructed by cloning a FLAG-tagged AIF mutant lacking amino acids 96 to 110 (AIFΔ96–110) into a cardiotropic AAV9 vector under the control of the cardiac troponin T (cTnT) promoter (VectorBuilder Inc., China). Both lentiviral and AAV9 vectors were produced by VectorBuilder Inc.

### Western blot and RT-qPCR

Total proteins were extracted from tissues or cultured cells using radioimmunoprecipitation assay lysis buffer supplemented with a mixture of phosphatase and protease inhibitors (Thermo Fisher Scientific, USA). Samples were then mixed with sodium dodecyl sulfate–PAGE sample loading buffer. Equal amounts of protein solution were loaded onto a polyacrylamide gel and electrophoresed. Proteins were subsequently transferred from the gel to a polyvinylidene difluoride membrane. Membranes were blocked with 5% bovine serum albumin for 1 h at 37 °C and incubated with the primary antibody overnight at 4 °C. The following day, membranes were incubated with fluorescent secondary antibodies and scanned using the Odyssey CLx 9140 Infrared Imaging System (LI-COR, USA). Protein bands were quantified using Image-Pro Plus software. The primary and secondary antibodies used in these experiments are listed in Table S2.

Total RNA was extracted from tissues or cells using TRIzol reagent (Invitrogen, USA), and complementary DNA was synthesized by the PrimeScript Reverse Transcription System (Takara, Japan). Then, RT-qPCR was performed using SYBR Green II (Takara, Japan). Primers were shown in Table S3.

### Molecular docking

The full-length AlphaFold-predicted structures of AIF and MTFR1L were retrieved from UniProt and used as receptor and ligand proteins, respectively. Protein–protein docking was performed using the HDOCK server, which samples all possible binding modes between 2 protein structures and ranks the predicted complexes based on a scoring function. To further characterize the binding interface, the Protein–Ligand Interaction Profiler (PLIP) online server was used to systematically identify and analyze specific interaction features within the protein–protein complexes. Additional interaction details were visualized and supplemented using PyMOL.

### Blood sampling and examination

Human plasma levels of MTFR1L were measured using human MTFR1L enzyme-linked immunosorbent assay kits purchased from Abbexa (catalog no. abx532866). Blood samples were collected from patients with AMI undergoing coronary PCI surgery and healthy controls in Peking University Third Hospital. This study complied with the Declaration of Helsinki and was approved by the Ethics Committee of Peking University Third Hospital.

### Statistical analysis

Data are expressed as the means ± SEM, and statistical analysis was performed with GraphPad Prism software. For comparisons between 2 groups, significance was determined using unpaired 2-tailed Student’s *t* test. For comparisons among multiple groups, 1-way or 2-way analysis of variance (ANOVA) followed by Tukey’s multiple comparisons was applied. Survival curves were assessed with the Kaplan–Meier method and compared by log-rank tests. The experimenters were blinded to animal genotype and grouping information, and all data were derived from biological replicates as indicated. Values of *P* < 0.05 were considered statistically significant.

## Ethical Approval

All animal procedures were approved by the Animal Welfare and Ethics Branch, Biomedical Ethics Committee of Peking University (ethical approval number: LA2020518).

## Data Availability

All data generated or analyzed during this study are included in this article and the Supplementary Materials or available from the corresponding author upon reasonable request.
