# Supplementary material for: Mitochondrial Fission Regulator 1-Like Protein Protects the Heart from Ischemia/Reperfusion Injury via Dual Mitochondrial Mechanisms
Source: Research (Wash D C). 2026 Apr 13;9:1241. doi: 10.34133/research.1241 (PMC13074272; doi:10.34133/research.1241)
Supplement: Supplementary 1 — Figs. S1 to S11 Tables S1 to S3 [file research.1241.f1.zip › Graphical abstract.docx]

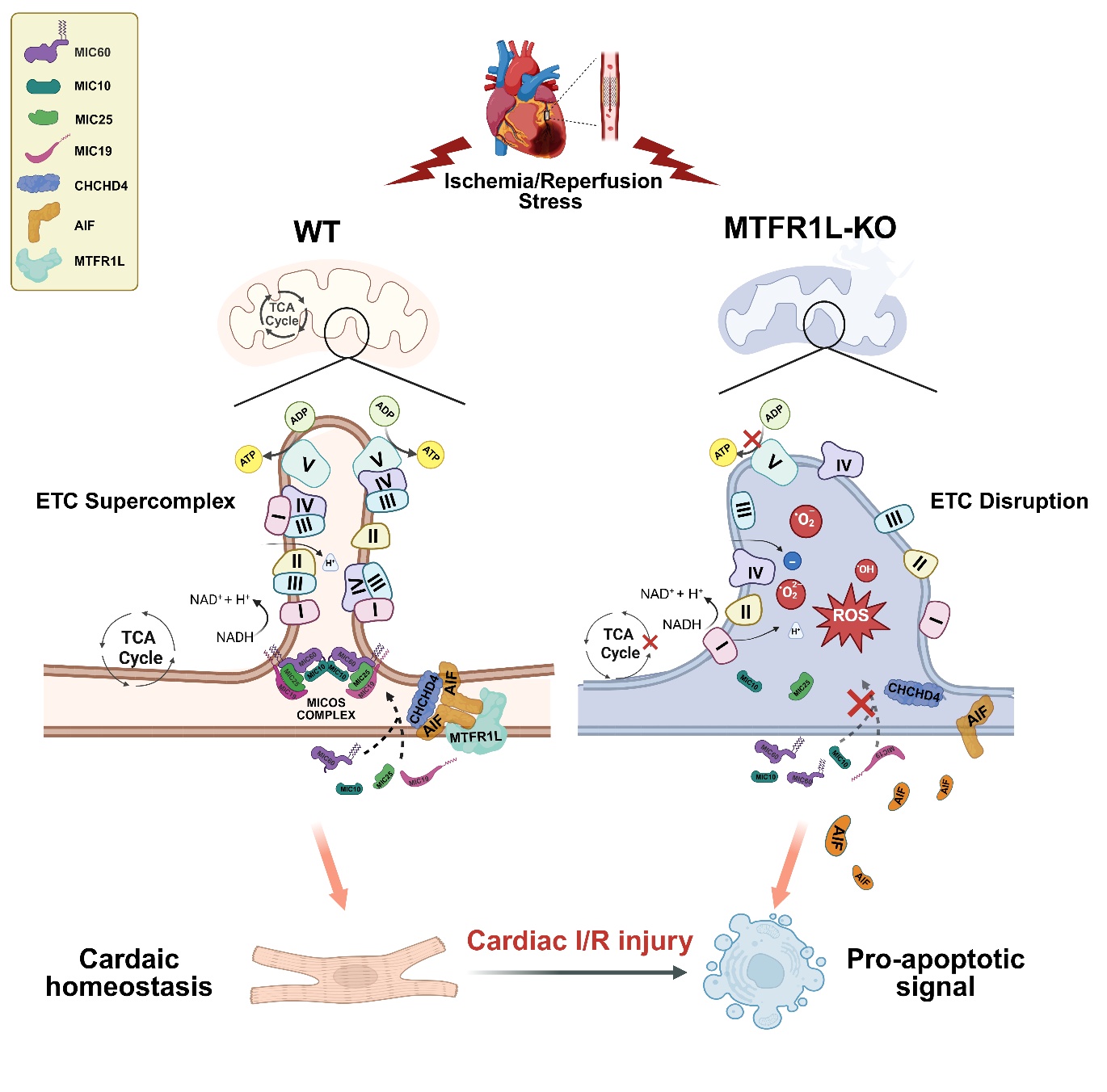


**Graphical abstract．Schematic diagram of the molecular mechanisms underlying MTFR1L- regulated cardiac I/R injury.**
