# Supplementary material for: Mitochondrial Fission Regulator 1-Like Protein Protects the Heart from Ischemia/Reperfusion Injury via Dual Mitochondrial Mechanisms
Source: Research (Wash D C). 2026 Apr 13;9:1241. doi: 10.34133/research.1241 (PMC13074272; doi:10.34133/research.1241)
Supplement: Supplementary 1 — Figs. S1 to S11 Tables S1 to S3 [file research.1241.f1.zip › Supplementary figures and legands minor revision.docx]

**Supplementary figures and legands**

**Supplementary Figure 1**

**
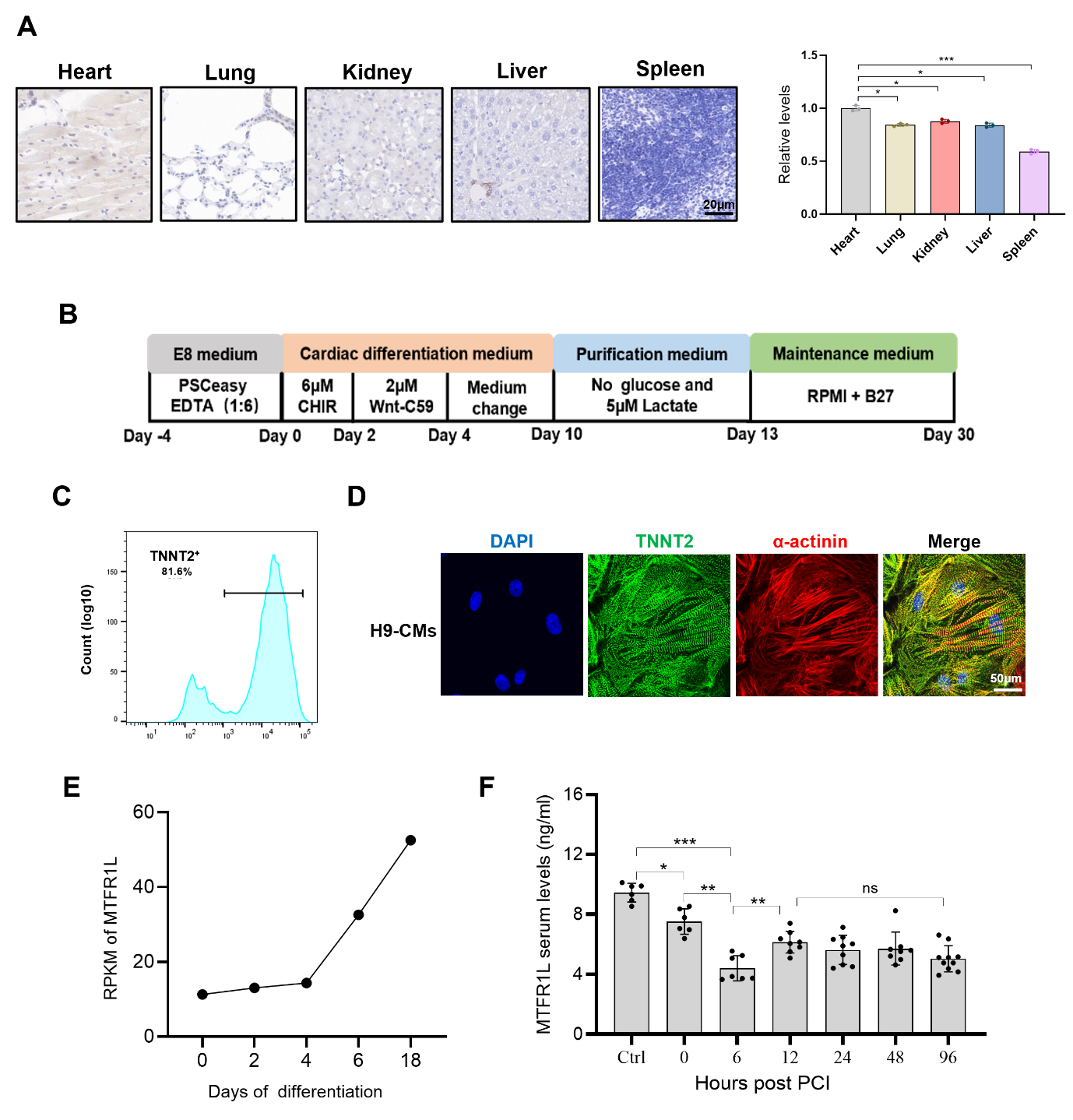
**

**Supplementary Figure 1. MTFR1L is highly expressed in mouse heart and human embryonic stem cells differentiated cardiomyocytes. A.** Immunohistochemical staining and quantitative analysis of MTFR1L in various mouse tissues, Scale bar = 20 μm. **B.** Schematic diagram outlining the differentiation process of H9 human embryonic stem cells into cardiomyocytes. **C.** Flow cytometry (FACS) analysis of H9-CMs, indicating high purity based on TNNT2 expression. **D.** Immunofluorescence staining of sarcomeric proteins (TNNT2 and α-actinin) confirming cardiac identity of H9-CMs, Scale bar = 50 μm. **E.** RNA-seq analysis demonstrating upregulation of MTFR1L mRNA during the differentiation of H9 cells into cardiomyocytes. **F.** Serum MTFR1L levels measured by immunoassay in acute ischemic myocardium (AIM) patients undergoing PCI surgery compared to healthy controls (Ctrl). *n* = 6-10 per group. Data are represented as mean ± SEM; *P<0.05, **P<0.01, ***P<0.001.Data presented in **F** were analyzed via one-way ANOVA followed by the Bonferroni post hoc test.

**Supplementary Figure 2**

**
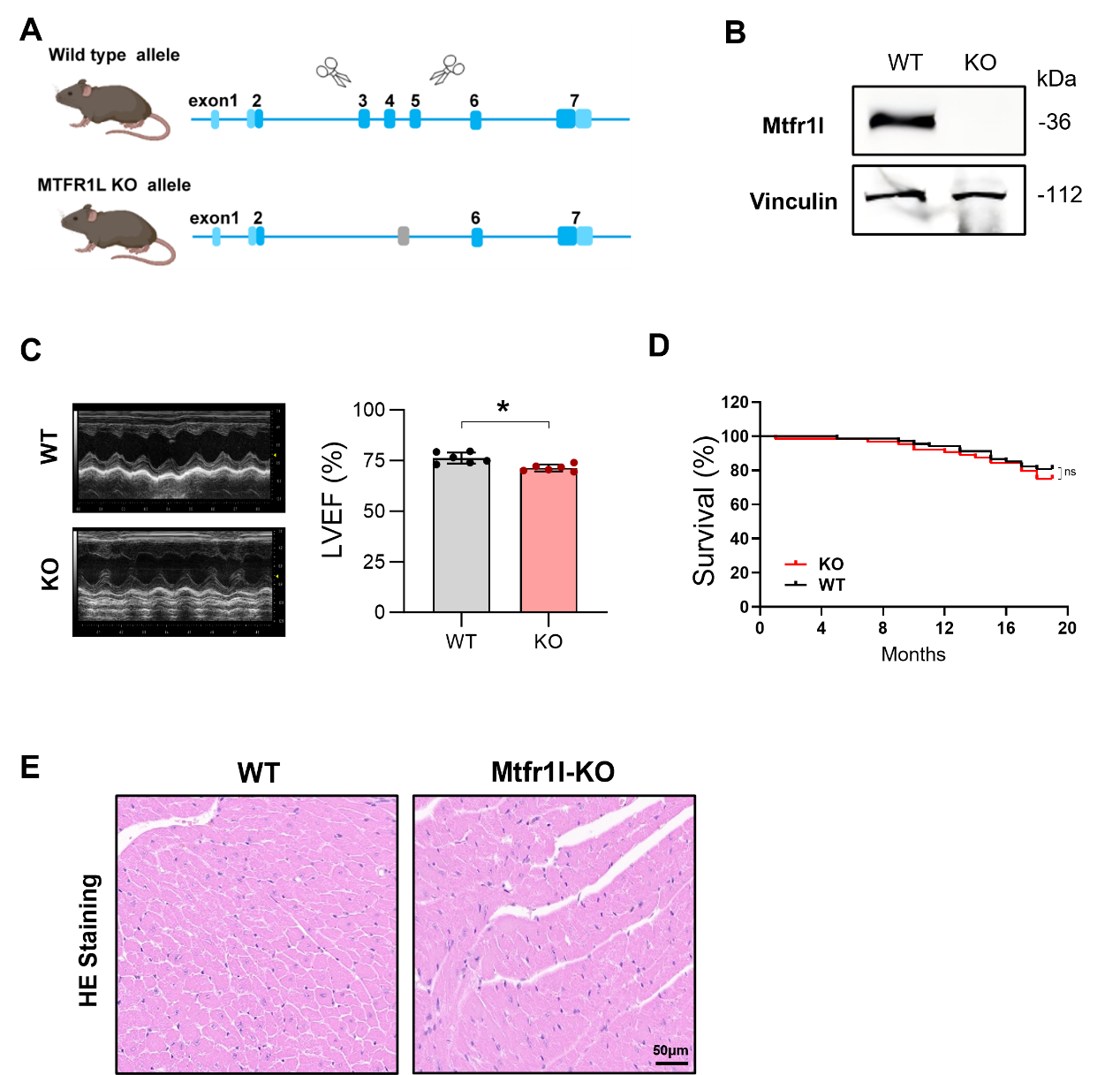
**

**Supplementary Figure 2. Characterization of Mtfr1l knockout mice under baseline conditions. A.** Schematic diagram illustrating the generation of Mtfr1l knockout mice using the CRISPR/Cas9 genome editing strategy. **B.** Western blot analysis confirming the absence of MTFR1L protein expression in Mtfr1l-KO mice. **C.** Representative echocardiographic imaging at 16 weeks and quantitative analysis of baseline cardiac function in Mtfr1l-KO versus WT mice. **D.** Kaplan–Meier survival curves comparing Mtfr1l-KO mice and wild-type (WT) littermates under physiological conditions. **E.** Representative hematoxylin and eosin (H&E) staining of heart tissue sections from Mtfr1l-KO and WT mice, showing normal myocardial histology in both groups, Scale bar = 50μm.

**Supplementary Figure 3**

**
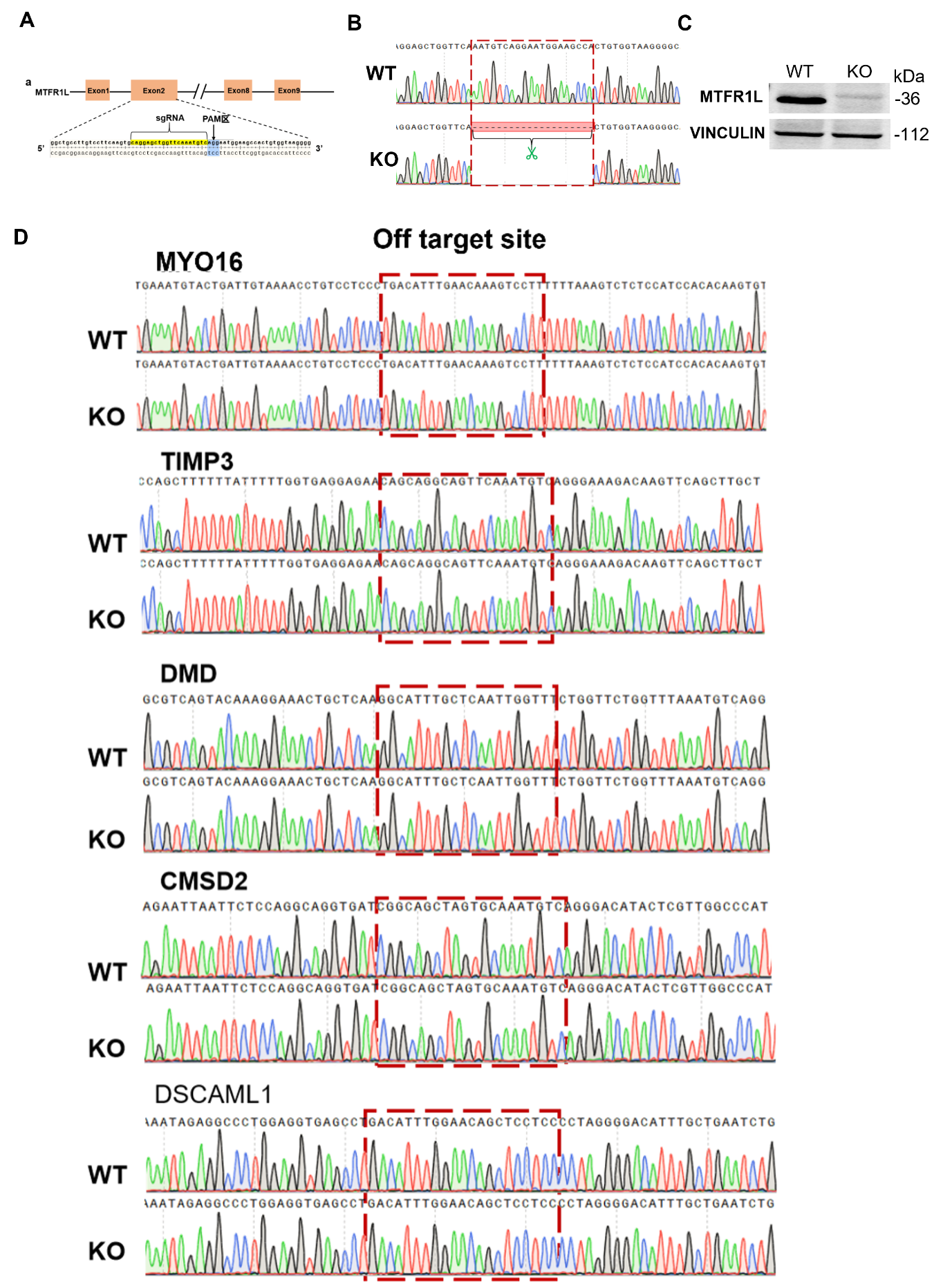
**

**Supplementary Figure 3. Generation of MTFR1L knockout models.** **A.** Schematic diagram of the CRISPR/Cas9 strategy used to generate MTFR1L knockout hESC. **B.** Sanger sequencing showing a 10-bp deletion introduced by spCas9 inH9-MTFR1L cells, resulting in a frameshift mutation. **C.** Western blot analysis confirming the absence of MTFR1L protein in knockout H9-CMs. **D.** Off-target analysis by Sanger sequencing in the top five predicted exon regions; no off-target mutations were detected.

**Supplementary Figure 4**

**
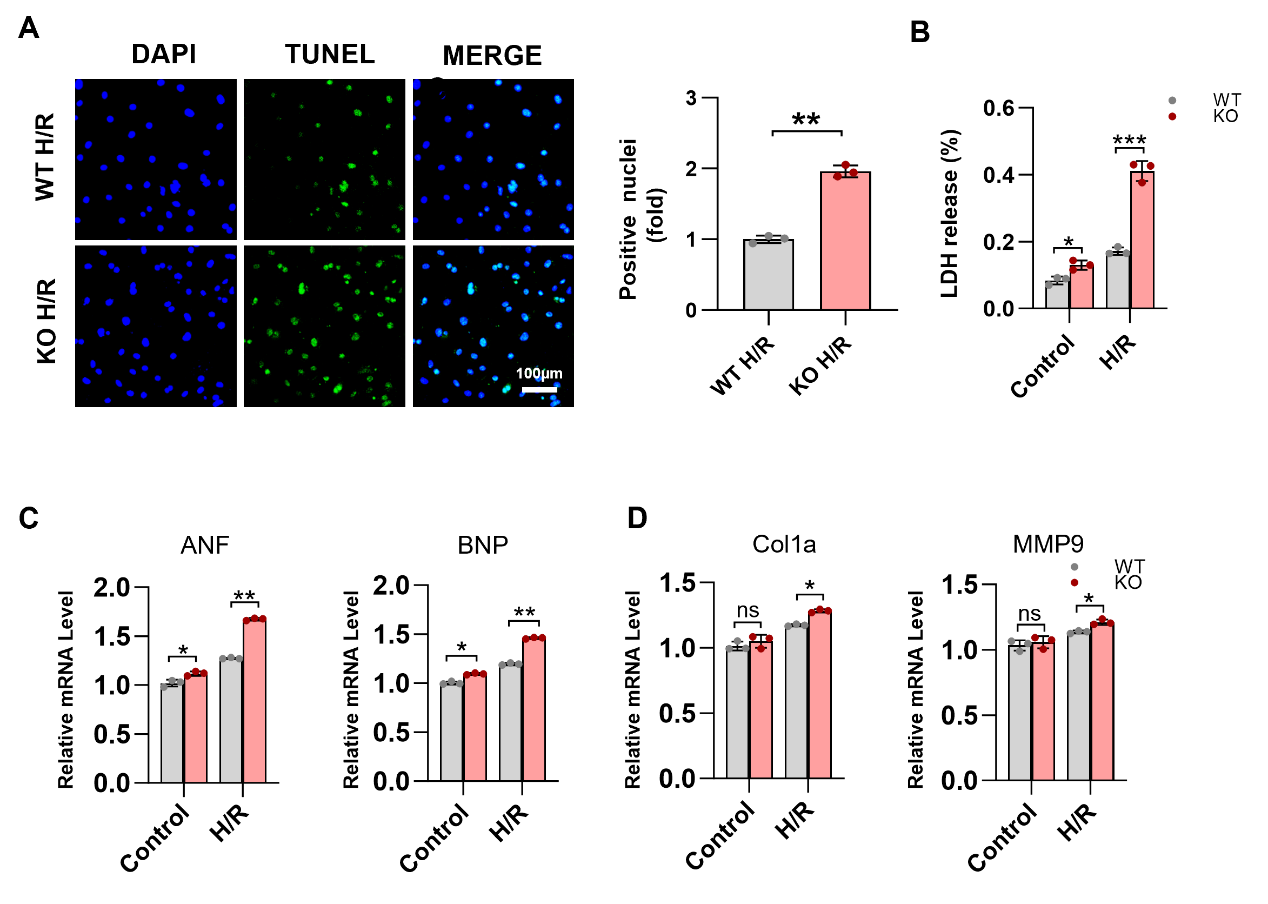
**

**Supplementary Figure 4. Loss of MTFR1L exacerbates H9-CMs H/R injury**. **A.** Representative immunofluorescence image and quantitative analysis of ROS levels in H9-derived MTFR1L-KO and WT cardiomyocytes following H/R treatment, Scale bar = 100μm. **B.** Measurement of LDH release in MTFR1L-KO and WT cardiomyocytes under H/R stress, n=3 per group. **C.** RT-qPCR analysis of canonical cardiac dysfunction markers in MTFR1L-KO and WT cardiomyocytes, n=3 per group. **D.** RT-qPCR analysis of fibrosis-related gene expression, n=3 per group. Data are represented as mean ± SEM; *P<0.05, **P<0.01, ***P<0.001. Data presented in **A** were analyzed via the Student *t* test. Data presented in **B-D** were analyzed via two-way ANOVA followed by the Bonferroni post hoc test.

**Supplementary Figure 5**


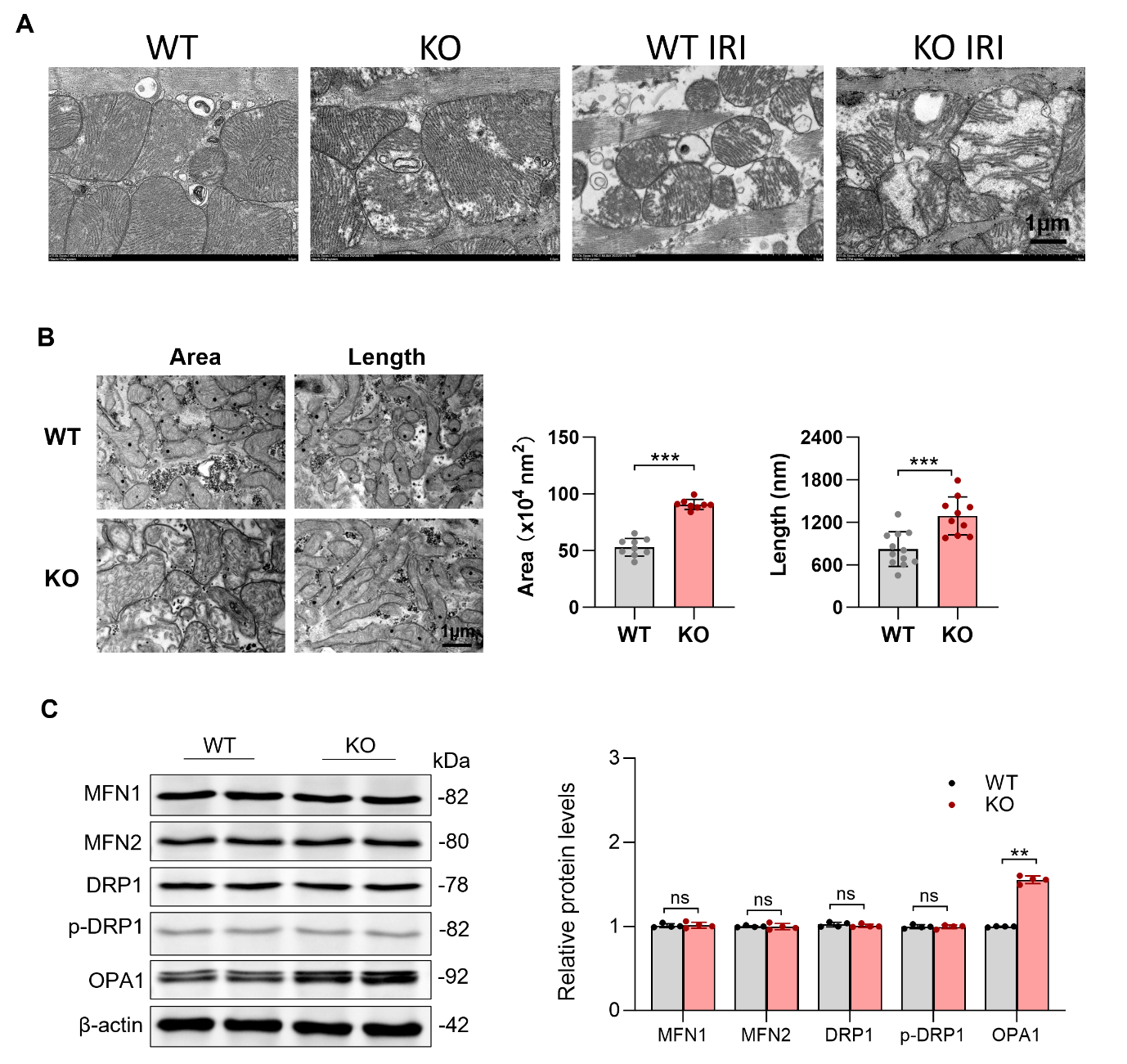


**Supplementary Figure 5 . Loss of MTFR1L alters mitochondrial structure. A.** Enlarged TEM images of mitochondrial ultrastructure in heart tissues from Mtfr1l-KO and WT mice under Sham conditions, corresponding to Figure 3A. Scale bar = 1 nm.. **B.**Transmission electron microscopy images and quantitative assessment of mitochondrial ultrastructure in MTFR1L-KO and WT cardiomyocytes derived from H9 cells，Scale bar = 200nm. **C.** Representative Western blot and statistical analysis of dynamic proteins in MTR1L-KO and WT H9-CMs, n = 4 per group. Data are represented as mean ± SEM; *P<0.05, **P<0.01, ***P<0.001. Data presented in **B, C** were analyzed via the Student *t* test.

**Supplementary Figure 6**

**
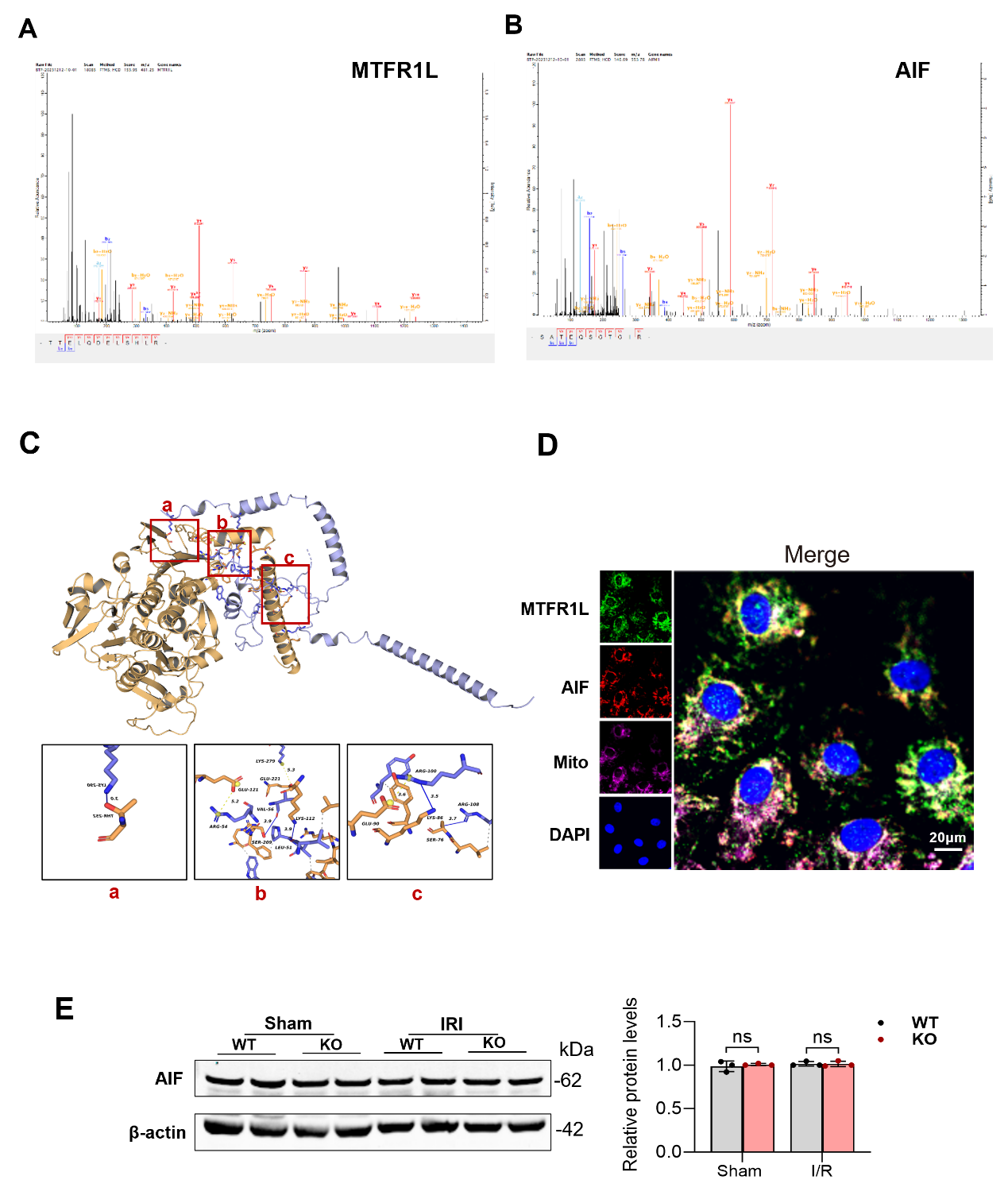
**

**Supplementary Figure 6. MTFR1L interacts with AIF as predicted by molecular docking. A-B.** Mass spectrometry identification of MTFR1L- and AIF-specific peptide segments. **C.** Predicted binding models of MTFR1L and AIF from molecular docking analysis: (a) Potential interaction between LYS-290 (MTFR1L) and THR-252 (AIF); (b) Potential binding between VAL-56 (MTFR1L) and SER-209 (AIF), or between LYS-112 (AIF) and LEU-51 (MTFR1L); (c) Interaction between ARG-100 (MTFR1L) and LYS-86 or SER-76 (AIF). D. Immunofluorescence staining showing co-localization of MTFR1L (green), AIF (red), and mitochondria (pink) in H9-CMs. Scale bar = 20 μm**. E.** Western blot analysis and quantification of total AIF levels in whole heart lysates from Mtfr1l-KO and WT mice under sham or I/R conditions，n=3 per group. Data are represented as mean ± SEM; *P<0.05, **P<0.01, ***P<0.001. Data presented in **E** were analyzed via two-way ANOVA followed by the Bonferroni post hoc test.

**Supplementary Figure 7**


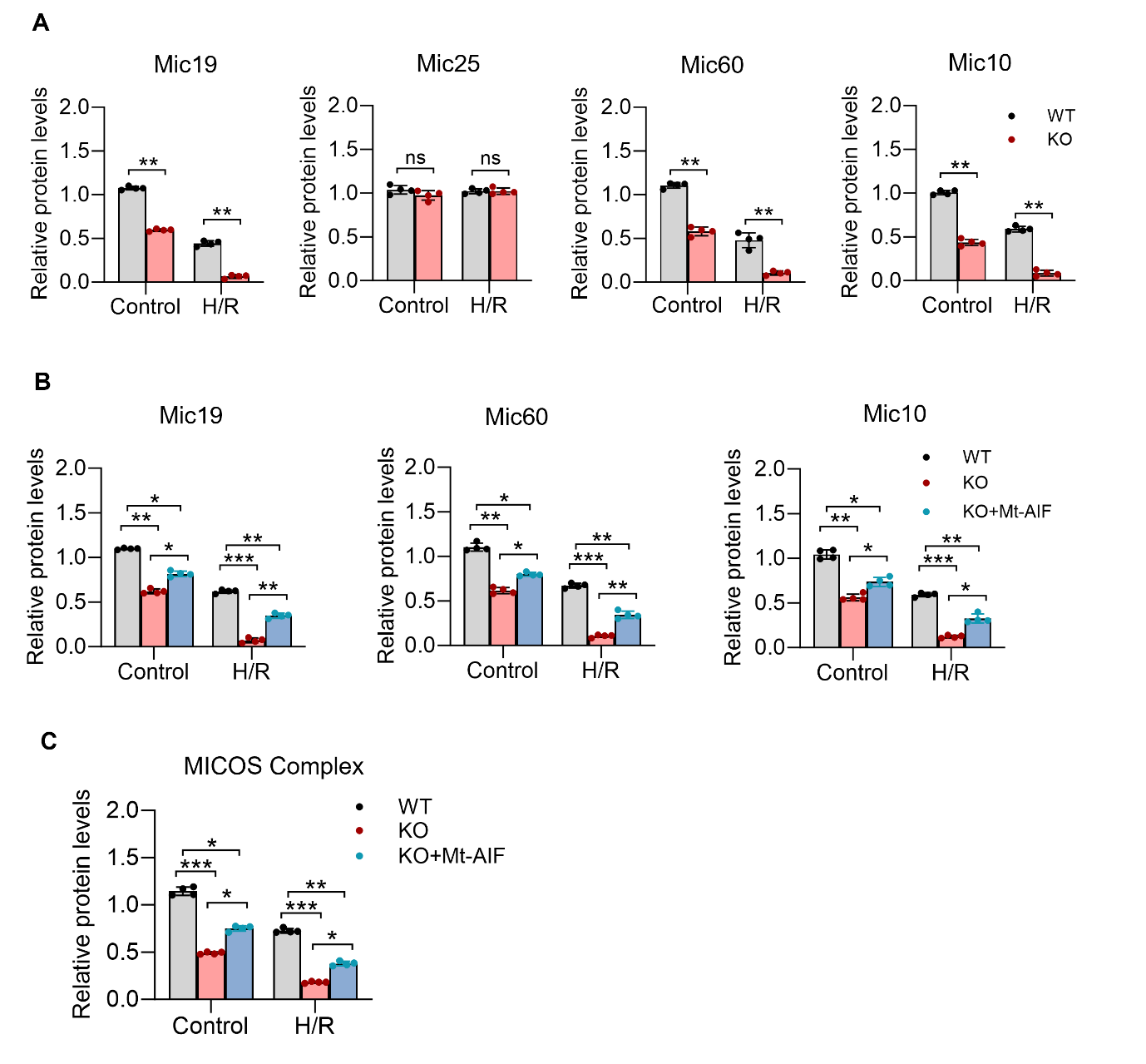


**Supplementary Figure 7. Quantification of MICOS complex and subunit protein levels. A.** Quantification of the levels of MICOS subunits in Figure 6D. n=4 sample per group. **B.** Quantification of the levels of MICOS subunits after Mt-AIF overexpression in Figure 6H, n=4 sample per group. **C.** Quantification of the levels of MICOS complex after Mt-AIF overexpression in Figure 6I, n=4 sample per group. Data are represented as mean ± SEM; *P<0.05, **P<0.01, ***P<0.001. Data presented were analyzed via two-way ANOVA followed by the Bonferroni post hoc test.

**Supplementary Figure 8**


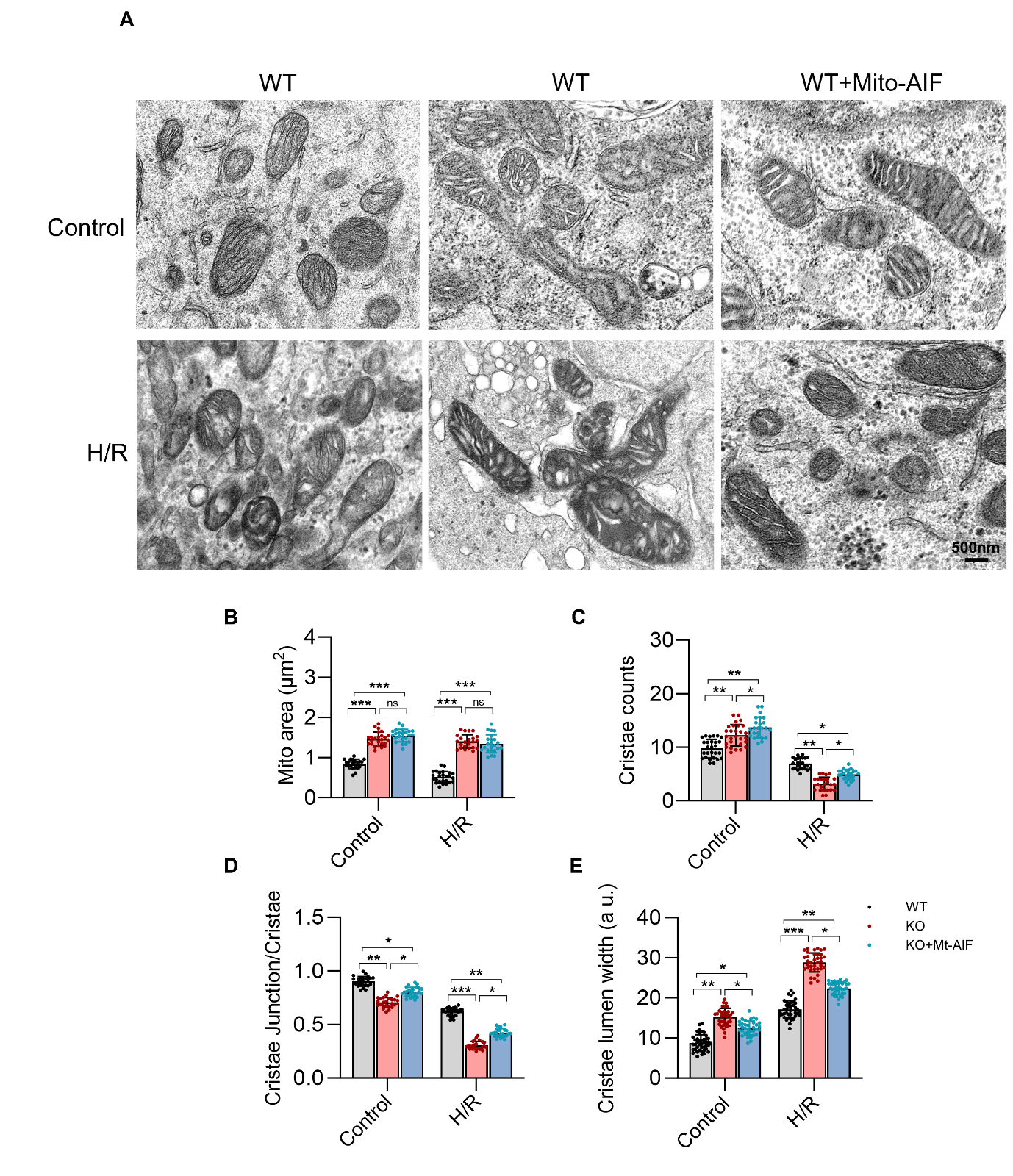


**Supplementary Figure 8. Mt-AIF overexpression in MTFR1L-KO H9-CMs restored mitochondrial cristae structure. A.**Representative transmission electron microscopy (TEM) images showing mitochondrial ultrastructure alternations in MTFR1L-KO **H9-CMs** after Mt-AIF overexpression**.** Scale bar = 500 μm. **B-E.** Quantification of mitochondrial ultrastructural parameters (including size, cristae junctions, cristae number, and lumen width), n=6 sample per group. Data are represented as mean ± SEM; *P<0.05, **P<0.01, ***P<0.001. Data presented were analyzed via two-way ANOVA followed by the Bonferroni post hoc test.

**Supplementary Figure 9**

**
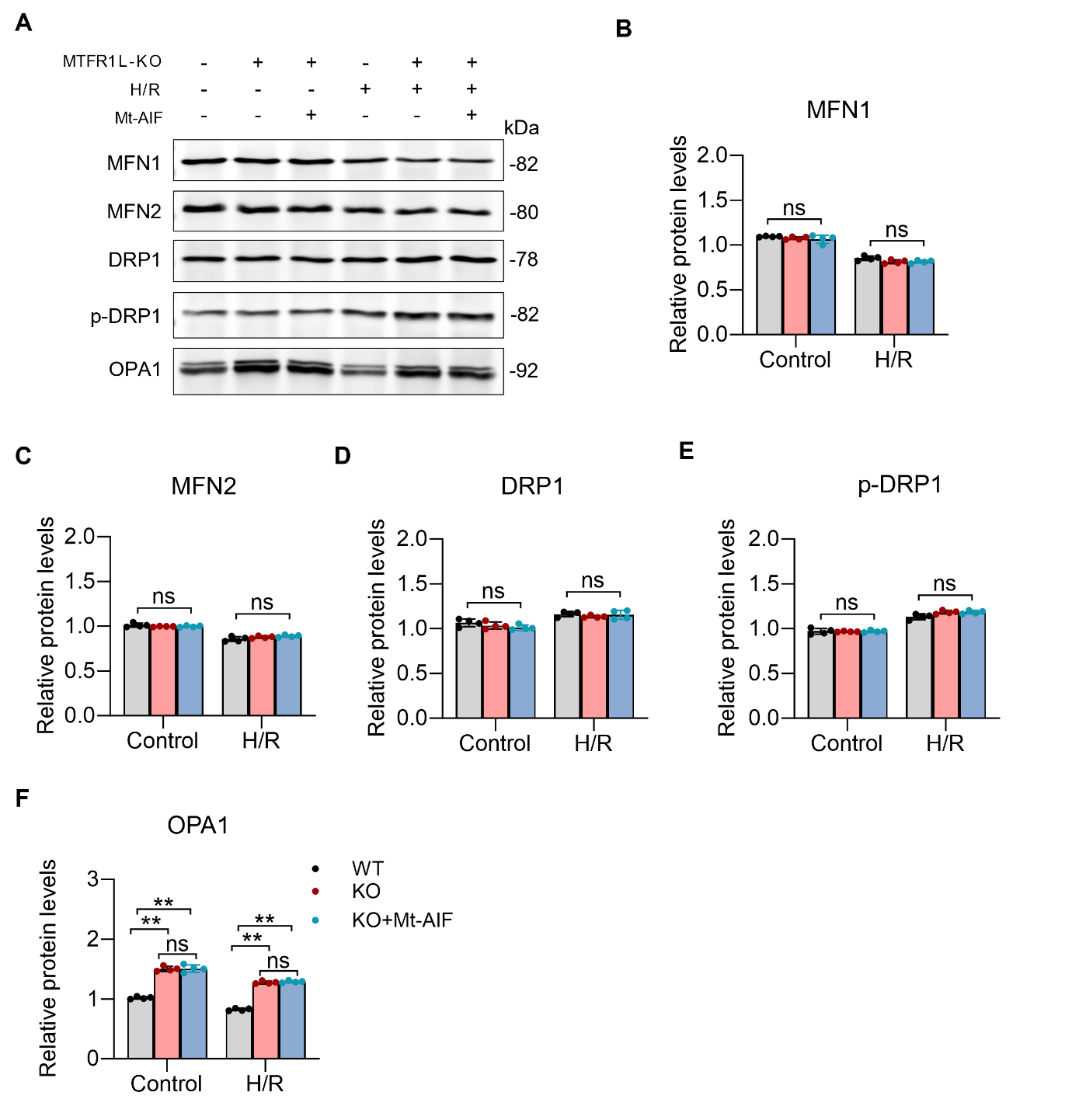
**

**Supplementary Figure 9. Mt-AIF overexpression did not affect mitochondrial dynamics. A.** Representative Western blot and statistical analysis of dynamic proteins in H9-CMs after Mt-AIF overexpression. **B-F.** Quantification of the levels of mitochondrial dynamic proteins after Mt-AIF overexpression in Supplementary Figure 9A , n=4 sample per group. Data are represented as mean ± SEM; *P<0.05, **P<0.01, ***P<0.001. Data presented were analyzed via two-way ANOVA followed by the Bonferroni post hoc test.

**Supplementary Figure 10**

**
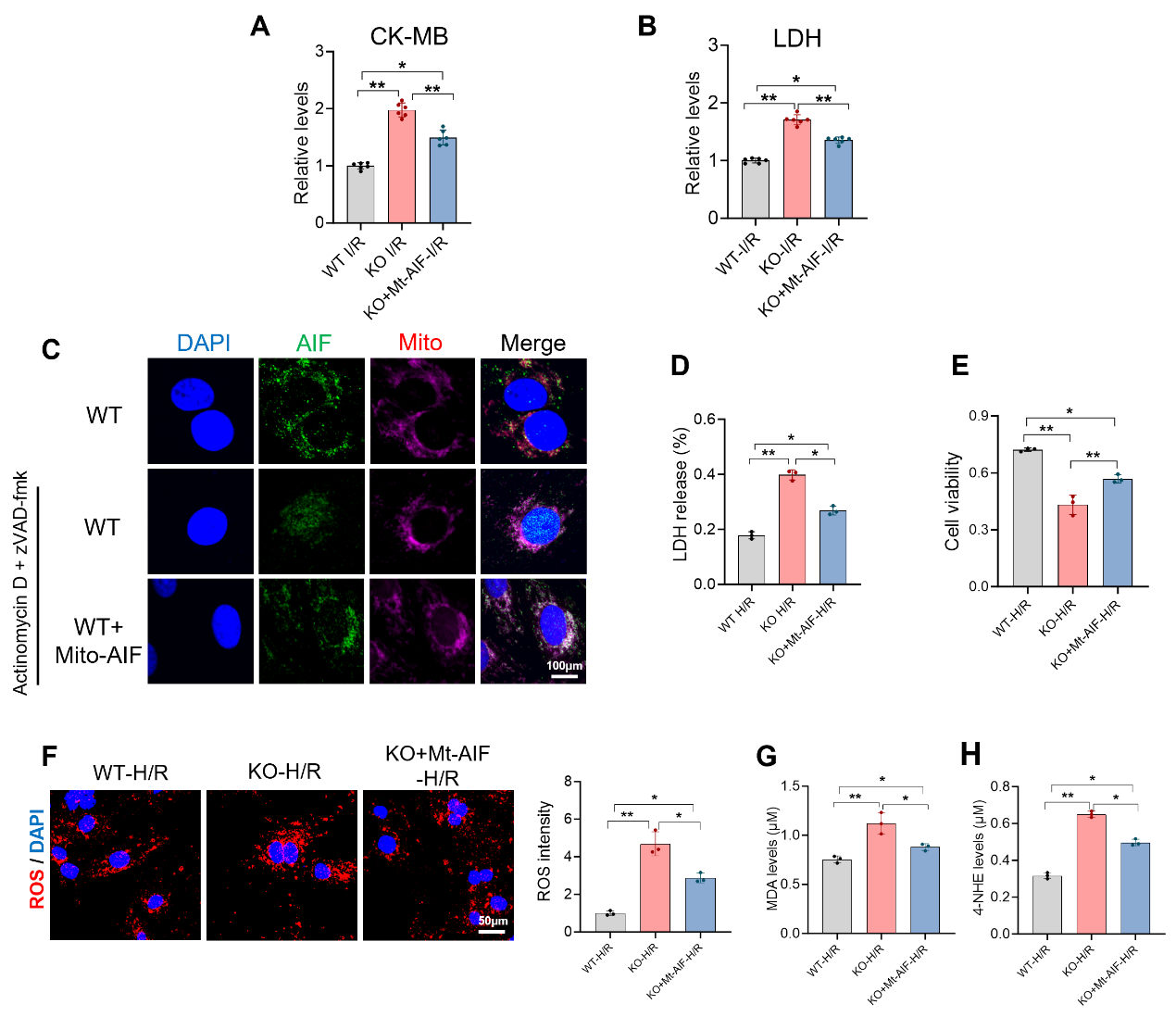
**

**Supplementary Figure 10. Mt-AIF overexpression alleviates ischemic injury in MTFR1L-deficient cardiomyocytes. A-B.** Serum levels of CK-MB and LDH released from Mtfr1l-KO mouse hearts with or without Mt-AIF overexpression following I/R injury, n=6 per group. **C.** Representative immunofluorescence images showing subcellular localization of Mt-AIF under actinomycin D and zVAD-fmk stimulation, Scale bar = 100 μm. **D.** H9-derived MTFR1L-KO cardiomyocytes with or without Mt-AIF overexpression following H/R treatment. n = 3 per group. n = 3 per group. **E.** Cell viability of H9-derived MTFR1L-KO cardiomyocytes with or without Mt-AIF overexpression following H/R treatment, n=3 per group.**F.** Representative ROS staining and quantitative analysis in H9-derived WT and MTFR1L-KO cardiomyocytes with or without Mt-AIF overexpression following H/R treatment. n = 3 per group, Scale bar = 50 μm. **G-H.** Quantification of oxidative stress byproducts (MDA, 4-HNE) assessed by immunoassay. n= 3 per group. Data are represented as mean ± SEM; *P<0.05, **P<0.01, ***P<0.001. Data presented in **A, B, D, E, G, H** were analyzed via one-way ANOVA followed by the Bonferroni post hoc test.

**Supplementary Table 1.** Top 20 interactors identified by Mass spectrometry

| No. | Protein description | Mito location scores |
| --- | --- | --- |
| 1 | 40S ribosomal protein S28 | ** |
| 2 | Glutamine--tRNA ligase | *** |
| 3 | Eukaryotic translation initiation factor 2 subunit 3 | Not predicted |
| 4 | Mitochondrial fission regulator 1-like | *** |
| 5 | DNA damage-binding protein 1 | ** |
| 6 | Acetyl-CoA acetyltransferase | **** |
| 7 | Putative ATP-dependent RNA helicase DHX30 | **** |
| 8 | Leucine--tRNA ligase | *** |
| 9 | Very-long-chain (3R)-3-hydroxyacyl-CoA dehydratase 3 | ** |
| 10 | Complement C3; Complement C3 beta | *** |
| 11 | DnaJ homolog subfamily A member 2 | ** |
| 12 | Pre-mRNA-processing-splicing factor 8 | ** |
| 13 | 116 kDa U5 small nuclear ribonucleoprotein component | * |
| 14 | 26S proteasome non-ATPase regulatory subunit 2 | ** |
| 15 | Eukaryotic translation initiation factor 3 subunit C | Not predicted |
| 16 | 60S acidic ribosomal protein P2 | ** |
| 17 | Mini-chromosome maintenance complex-binding protein | * |
| 18 | Apoptosis-inducing factor 1 | **** |
| 19 | Coatomer subunit alpha; Xenin; Proxenin | * |
| 20 | DNA replication licensing factor MCM2 | ** |

Mitochondrial location probability was predicted using the COMPARTMENTS protein subcellular database (https://compartments.jensenlab.org), with confidence scores ranging from * (low) to ***** (high). ‘Not predicted’ indicates absence of a detectable mitochondrial localization signal. The database integrates four scoring methods, and the highest score among them was selected in this table for each protein.

**Supplementary Table 2.** **Antibodies used in this study**

| **Name** | **RRID** |
| --- | --- |
| Anti-MTFR1L | (Atlas Antibodies Cat# HPA027124, RRID:AB_10600211) |
| Anti-GAPDH | (Abcam Cat# ab8245, RRID:AB_2107448) |
| Anti-FLAG | (Cell Signaling Technology Cat# 14793, RRID:AB_2572291) |
| Anti-Vinculin | (Santa Cruz Biotechnology Cat# sc-73264 RRID:AB_1131292) |
| Anti-β-actin | (Yeasen Biotech Cat# 30101ES, RRID:AB_3644235) |
| Anti-VDAC1 | (Cell Signaling Technology Cat# 4866, RRID:AB_2272627) |
| Anti-AIF | (Cell Signaling Technology Cat# 4642, RRID:AB_2224542) |
| Anti-Lamin B | (Proteintech Cat# 66095-1-Ig, RRID:AB_11232208) |
| Anti-cTNT | (Abcam Cat# ab8295, RRID:AB_306445) |
| Anti-α-actinin | (Abcam Cat# ab68194, RRID:AB_3064857) |
| Anti-DRP1 | (Cell Signaling Technology Cat# 8570,  RRID：AB_109504) |
| Anti-p-DRP1 | (Cell Signaling Technology Cat# 3455, RRID:AB_2085352) |
| Anti-OPA1 | (Proteintech Cat# 27733-1-AP, RRID: AB_2810292) |
| Anti-MFN1 | (Cell Signaling Technology Cat# 14739, RRID:AB_2744531) |
| Anti-MFN2 | (Cell Signaling Technology Cat# 11925, RRID: AB_2750893) |
| Anti-MIC60 | (Proteintech Cat# 10179-1-AP, RRID: AB_2127193) |
| Anti-MIC25 | (Proteintech Cat# 20639-1-AP, RRID: AB_10697667) |
| Anti-MIC19 | (Proteintech Cat# 10179-1-AP, RRID: AB_2127193) |
| Anti-MIC10 | (Proteintech Cat# 25625-1-AP, RRID: AB_2687533) |
| Anti-CHCHD4 | (Proteintech Cat# 21090-1-AP, RRID: AB_10734583) |
| Total OXPHOS Rodent Cocktail | (Abcam Cat# ab110413, RRID:AB_2629281) |
| Goat anti-Mouse IgG Alexa Fluor 594 | (Thermo Fisher Scientific Cat# A-11005, RRID:AB_2534073) |
| Goat anti-Mouse IgG Alexa Fluor 488 | (Thermo Fisher Scientific Cat# A-11001, RRID:AB_2534069) |
| Goat anti-Rabbit IgG Alexa Fluor 594 | (Thermo Fisher Scientific Cat# A-11012, RRID:AB_2534079) |
| Goat anti-Rabbit IgG Alexa Fluor 488 | (Thermo Fisher Scientific Cat# A-11008, RRID:AB_143165) |
| IRDyeR 800CW Goat anti-Rabbit IgG(H+L) | (Li-Cor Biosciences, Cat# 925-32211, RRID: AB_2651127) |
| IRDyeR 800CW Goat anti-Mouse IgG(H+L) | (Li-Cor Biosciences, Cat# 926-32210, RRID: AB_621842) |

**Supplementary Table 3. Primers and sequences used in this study**

| **Gene** | **Forward (5’-3’)** | **Reverse (5’-3’)** |
| --- | --- | --- |
| Nd1 (Mouse) | GGCCCATTCGCGTTATTCTT | GTATGGTGGTACTCCCGCTG |
| Nd2 (Mouse) | TTCGTCACACAAGCAACAGC | AGTAGAGTTGAGTAGCGGGT |
| Nd3 (Mouse) | AGTTGCATTCTGACTCCCCC | CTTGTAGGGTCGAATCCGCA |
| Nd4 (Mouse) | TCCTCAGACCCCCTATCCAC | AATCCCTGCGTTTAGGCGTT |
| Nd5 (Mouse) | CAACGCCTGAGCCCTACTAA | AGGACTGGAATGCTGGTTGG |
| Sdha (Mouse) | GGAAGATTACAAAGTGCGGGTCGAT | GGTCTTGTCGATAACAGGTCTG |
| Sdhb (Mouse) | GCTGGATGGTGGTGGTATGTG | CGTGCTTGGTGGTCATGTTG |
| Sdhd (Mouse) | TGGGAAGACTGGGAAGACTG | CGGTGGTGGTAATGTGGATG |
| Sdhaf1 (Mouse) | GCTGGATGGTGGTGGTATGTG | CGTGCTTGGTGGTCATGTTG |
| Cytb (Mouse) | AATCCACTAAACACCCCACCC | AGTCGGCATCGTTTATGGTC |
| Cox1 (Mouse) | TCCCTTGACATCGTGCTTCA | AGTCTGAGTAGCGTCGTGGT |
| Cox2 (Mouse) | TCTACAAGACGCCACATCCC | ACGGGGTTGTTGATTTCGTCT |
| Cox3 (Mouse) | ACCCTTGGCCTACTCACCAA | GCAGCCTCCTAGATCATGTGT |
| Atp6 (Mouse) | TTCCCATCCTCAAAACGCCTAA | GAAGTGGGCAAGTGAGCTTTT |
| Atp8 (Mouse) | GCCACAACTAGATACATCAACATGA | ATTGTTGGGGTAATGAATGAGGCA |
| Col1α1(Mouse) | ACCTGCTAGACCACCTGGAG | CCTTGGCTGTTATCTTCGGTACCGG |
| Tgf-β1 (Mouse) | TGCGCTTGCAGAGATTAAAA | CGTCAAAAGACAGCCACTCA |
| Col3α1 (Mouse) | TGAAGGCGAATTCAAGGCTGAAGG | AGGGCCAATGTCCACACCAAATTC |
| BNP (human) | TCTGGCTGCTTTGGGAGGAAGA | CCTTGTGGAATCAGAAGCAGGTG |
| Col1α1 (human) | GATTCCCTGGACCTAAAGGTGC | AGCCTCTCCATCTTTGCCAGCA |
| MMP9 (human) | AGACCTGGGCAGATTCCAAAC | CGGCAAGTCTTCCGAGTAGT |
